# Satellites Detect Abatable Super-Emissions in One of the World’s Largest Methane Hotspot Regions

**DOI:** 10.1021/acs.est.1c04873

**Published:** 2022-02-01

**Authors:** Itziar Irakulis-Loitxate, Luis Guanter, Joannes D. Maasakkers, Daniel Zavala-Araiza, Ilse Aben

**Affiliations:** † Research Institute of Water and Environmental Engineering (IIAMA), Universitat Politècnica de València (UPV), Valencia 46022, Spain; ‡ SRON Netherlands Institute for Space Research, Utrecht 3584 CA, The Netherlands; § Environmental Defense Fund, Reguliersgracht 79, Amsterdam 1017 LN, The Netherlands; ∥ Institute for Marine and Atmospheric Research Utrecht, Utrecht University, Utrecht 3584 CC, The Netherlands

**Keywords:** methane emissions, plume detection and quantification, temporal monitoring, high-resolution satellite data, Turkmenistan, oil and gas

## Abstract

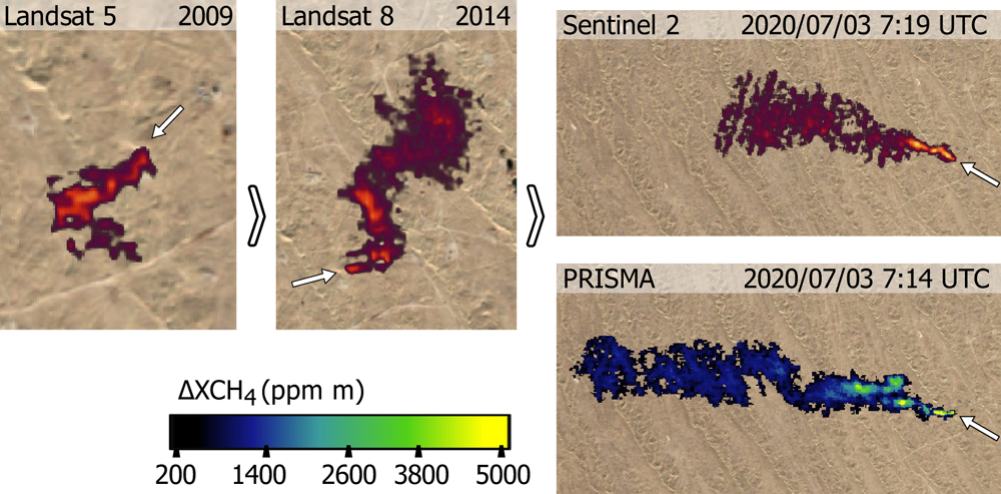

Reduction of fossil fuel-related methane emissions has been identified as an essential means for climate change mitigation, but emission source identification remains elusive for most oil and gas production basins in the world. We combine three complementary satellite data sets to survey single methane emission sources on the west coast of Turkmenistan, one of the largest methane hotspots in the world. We found 29 different emitters, with emission rates >1800 kg/h, active in the 2017–2020 time period, although older satellite data show that this type of emission has been occurring for decades. We find that all sources are linked to extraction fields mainly dedicated to crude oil production, where 24 of them are inactive flares venting gas. The analysis of time series suggests a causal relationship between the decrease in flaring and the increase in venting. At the regional level, 2020 shows a substantial increase in the number of methane plume detections concerning previous years. Our results suggest that these large venting point sources represent a key mitigation opportunity as they emanate from human-controlled facilities, and that new satellite methods promise a revolution in the detection and monitoring of methane point emissions worldwide.

## Abstract 2

New satellite methods allow to identify 29 super-emitters in Turkmenistan, most of them linked to inactive flaring venting massive amounts of gas.

## Introduction

Methane (CH_4_) is the second most important anthropogenic greenhouse gas, with a relatively short lifetime in the atmosphere (9 ± 1 years) and with 86 times the global warming potential of carbon dioxide over 20 years.^1^ During the past few decades, CH_4_ concentrations have risen rapidly^2^ to record highs that compromise the 2 °C temperature target of the Paris Agreement relative to the preindustrial era.^3^ Therefore, the reduction of CH_4_ emissions has been identified as a key climate change mitigation measure in the short to medium term.^4^

Among the sectors with the highest contributions to CH_4_ emissions is the oil and gas (O&G) industry. CH_4_ emissions from this sector are particularly difficult to quantify because they are often the result of unplanned occurrences, i.e., leaks, equipment malfunctions, or abnormal process conditions, of which quantity, duration, and frequency can differ strongly across regions, operators, and stages of the O&G supply chain.^5^ These events can result in so-called super-emissions, which disproportionately account for a significant fraction of total emissions.^6−9^ In addition to unforeseen events, emissions from the sector can come from controlled flaring and venting processes, which are, respectively, the combustion and direct liberation of excess natural gas produced. Flaring and venting are primarily done for safety reasons,^10^ but may also be for economic or operational reasons.^11^ The objective of flaring is to avoid the direct release of gas in the atmosphere by burning it. However, numerous studies show that the use of flaring does not always guarantee complete combustion of the gas stream in the flare.^12−15^ Although the use of flaring is preferable to venting from climate perspective, both are seen as indicators of poor resource utilization, where the use of more economically and environmentally sustainable alternatives for the use of excess gas is preferred.^16^ The use and regulation of flaring and venting depend on the policies and laws in force in each country or region.^16,17^ Therefore, the credibility of globally reported industrial CH_4_ emissions has recently been highly questioned.^5^ The IEA (International Energy Agency) Methane Tracker report^18^ and the U.N.’s Global Methane Assessment^4^ conclude that a large fraction of the emission mitigation options are technically feasible and cost-effective, and that O&G companies can take considerable low-cost and cost-saving measures to reduce CH_4_ emissions from pipelines, drilling and other facilities, but this would require greater control of all phases of O&G extraction, processing, and transport.

Traditionally, the detection and measurement of emissions have been performed through onsite campaigns focusing on locations where suspected undeclared emissions may be present. In situ measurements of ground-based campaigns can be very costly and, depending on their objective, the data collected will be different. Airborne campaigns allow coverage of larger areas, but they can be expensive and not very practical in some cases. In this context, satellites are capable of emission detection and monitoring at different scales (from local to global) and over long periods of time. However, detection from space will be limited to large emissions.

Since 2017, the TROPOMI sensor onboard Sentinel-5P provides daily global CH_4_ concentration data with a 7 × 5.5 km^2^ pixel resolution.^19^ This allows detection of CH_4_ concentration enhancements at the regional scale (e.g.,^20−24^), but in general, does not enable the determination of single point sources. On the other hand, the GHGSat instruments and so-called hyperspectral satellite missions like PRISMA, ZY1 AHSI and Gaofen-5 AHSI can map CH_4_ plumes from single emitters at high spatial resolution (25–50 m GHGSat and 30 m the rest) with a detection limit roughly between 100 and 1000 kg/h, suitable to detect medium-to-strong point emitters worldwide.^13,25,26^ The systematic application of these measurements, however, is limited by their sparse spatio-temporal coverage (see Materials and Methods section). The recent realization of the CH_4_ mapping potential of so-called multispectral missions with frequent global coverage holds promise to alleviate this gap.^27^ Missions like Sentinel-2 (S2) and Landsat 8 (L8) cover the entire world with a relatively high spatial and temporal resolution (see Materials and Methods section and Table S1), so they can continuously monitor CH_4_ plumes under favorable conditions (typically, strong emissions over spatially homogeneous areas). This recently developed satellite-based CH_4_ monitoring scenario allows to detect single point emissions of the largest CH_4_ hotspot regions in the world, which are identified with TROPOMI’s moderate resolution observations.^28^

**Figure 1:**
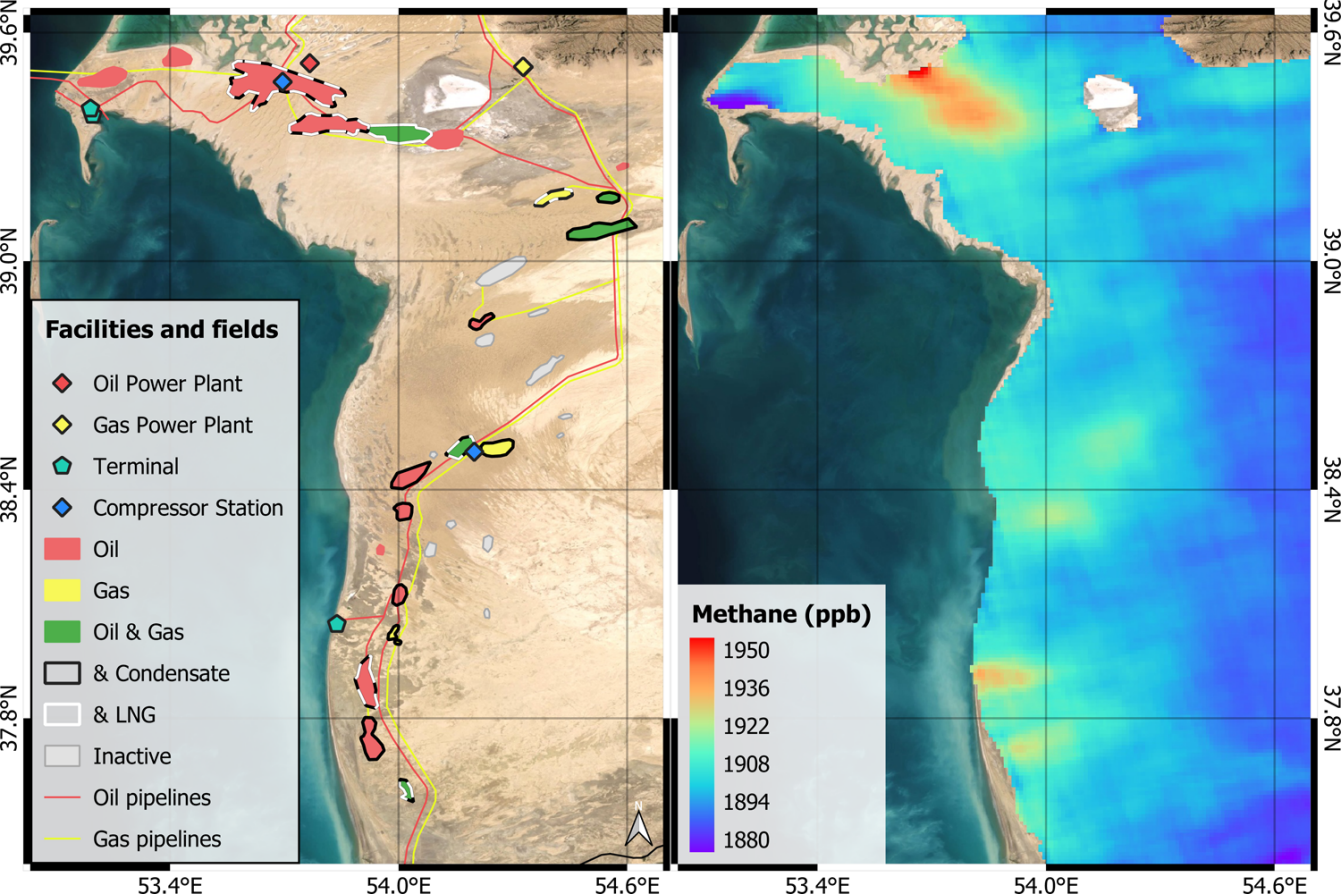
Representation of the study area. Left, oil and gas fields classified according to the type of production activity based on Rystad database:^31^ oil, gas, condensate, liquefied natural gas (LNG), and the combination of several of them; the location of processing plants, terminals, compressor stations, and pipelines along the South Caspian Basin as provided in^32^ are also depicted. Right, 0.1° composite of CH_4_ concentration in the atmospheric column from TROPOMI data between November 2018 and November 2020. Background satellite image from ESRI.

One example of those CH_4_ hotspot regions is the west coast of Turkmenistan, located in the Balkan province on the shores of the Caspian Sea, within the South Caspian Basin (SCB). This is a desert area where the main human activity is the production of O&G and derived products, and an abundant presence of mud volcanoes (more than 20), some of which are associated with O&G seepage.^29^ According to Scarpelli et al.,^30^ the country of Turkmenistan is one of the largest emitters of CH_4_ from O&G-related sources: eighth in oil-derived emissions (0.88 Tg a^–1^) and ninth in gas emissions (0.52 Tg a^–1^) in 2016, although the IEA estimates a total of 3.9 Tg a^–1^ of CH_4_ emissions in 2020 (almost three times more).^18^ In recent years TROPOMI has detected strong CH_4_ concentration enhancements in the western coastal belt belonging to the SCB. In this region there are 26 active fields, 21 onshore and 5 offshore, producing crude oil, condensate, liquefied natural gas (LNG), and gas in different proportions (see Figure 1).

In this work, we generate a satellite-based high spatial and temporal resolution survey of CH_4_ point emissions over the west coast of Turkmenistan based on the hotspot locations provided by the TROPOMI observations. This survey covers an area of approximately 21,500 km^2^ and the time period between January 2017 and November 2020. Our analysis relies on three different types of space-based CH_4_ measurements, which are used synergistically: TROPOMI data facilitate the delimitation of the study area and the identification of the most active regions; the hyperspectral images from PRISMA and ZY1 AHSI allow the identification of medium-to-strong emitters and the accurate quantification of emission rates for those regions in a limited set of days; finally, the multispectral data from S2 and Landsat enable the constant monitoring of the emissions from the emission points unveiled by the hyperspectral data (see the Materials and Methods section). We choose the west coast of Turkmenistan for this study because it offers an ideal combination of extreme CH_4_ emissions with a bright and relatively homogeneous surface. This allows us to best evaluate this unprecedented combination of CH_4_ data streams as well as to extract its full potential.

## Materials and Methods

### Definition of the Study Area with TROPOMI XCH_4_ Data

The TROPOspheric Monitoring Instrument (TROPOMI) sensor onboard ESA’s Sentinel-5P satellite^19^ provides daily global coverage of CH_4_ data with 7 km × 7 km (since August 2019, 5.5 km × 7 km) pixel resolution in nadir that allows finding areas with high CH_4_ concentration enhancements (find more technical information in the Table S1). The approximate location of the strongest sources in the study area has been identified using the wind rotation method introduced by Maasakkers et al.^28^ After identification of an area with large CH_4_ concentrations, data from individual days is rotated around a possible target point using the wind direction at the location. In this manner, the scenes are rotated so that the wind vector is always pointing northward, these rotated scenes are then averaged. By doing these exercises for a full grid of points, the location can be determined where the mean downwind concentrations are most significantly enhanced compared to the mean upwind concentrations, resulting in the most likely location of the source.^28^ TROPOMI pinpointing identified five key points (see Figure S1) where we started the search for point sources of emission. In addition, the Korpeje area was already known for its strong and frequent point source emissions.^25^

### High-Resolution Hyperspectral and Multispectral Data

This study has used both hyperspectral and multispectral satellites, which are complementary in the detection and monitoring of CH_4_ emissions. Hyperspectral instruments offer higher sensitivity to CH_4_ thanks to tens of spectral channels located around the strong CH_4_ absorption feature around 2300 nm, but acquisitions are upon request, and their coverage is sparse in space and time. In turn, multispectral systems provide frequent and spatially continuous observations over any region on Earth but with lower sensitivity to CH_4._

For this study, we have collected data from the ZY1 AHSI and PRISMA missions (see Table S1). Those are the only two hyperspectral satellite missions sampling the 2300 nm spectral region with an open data policy. In total, we have obtained 12 images from PRISMA and one from ZY1, all of them acquired during 2020 (the last year covered by this study). See Figure S2 for more information.

The hyperspectral images have allowed us to observe CH_4_ emissions with 30 m spatial resolution and quantify the emissions using the matched filter method.^13^ The quantification has been done with the integrated mass enhancement (IME) method,^33^ and we have used 1-h average 10-m wind (*U*10) data from the NASA Goddard Earth Observing System-Fast Processing (GEOS-FP) meteorological reanalysis product at 0.25° × 0.3125° resolution^34^ to get the Flux Rates (Q). The details of our processing of hyperspectral data are provided in ref (35).

For the temporal monitoring of emissions, we have used the multispectral Sentinel-2 (S2) mission. We have chosen the S2 Level 2A (L2A) product from both S2-A and B satellites of the ESA Copernicus program, which provides 20 m pixel resolution data in B11 and B12 bands with less than 5 days revisit time (see Table S1). This data is openly available on the Copernicus Open Access Hub official portal.

We have defined the S2 CH_4_ detection limit and the estimation of the emissions detected in S2 monitoring using the quantified hyperspectral plumes coincident with S2 detections, as the three satellites have approximately the same overpass time with a few minutes difference (between 2 and 5) in the observations used. We have identified nine simultaneous plumes indicating that S2 can detect plumes that maintain CH_4_ concentrations above ∼3800 ppm m. The minimum flux rate we have estimated from this data is 1800 kg/h in a pit flare emission with a wind speed of 0.28 m/s (see Figure S3). We set this value as the detection limit. However, it should be noted that this limit may vary for other surfaces in the area and other wind speeds.

The detection of single plumes from S2 data is often challenging because of its lower sensitivity to CH_4_ concentration enhancements. We have a priori predetermined areas with potential emitters on which to focus the search of possible plumes. These are the areas near the TROPOMI pinpoints (see Figure S1), emission points detected in the ZY1 and PRISMA hyperspectral images (see Figure S4), O&G extraction fields in the SCB according to refs (31, 32), pipeline crossings, flares that in the past had shown an active flame, and mud volcanoes.

To detect CH_4_ emissions with S2, we have used a similar methodology described by Varon et al.^27^ as multiband/single-pass (MBSP), combined with a dynamic multitemporal method. To do that, we have applied the B12/B11 band ratio to all clear-sky day observations of both the S2A and S2B satellites using the timelapse tool provided in the online service EO Browser of Sentinel Hub.^36^ In this way, we have obtained the continuous record of the time series of the study area (<3 km^2^ in each timelapse). We have discarded cloudy images with an automatic filter available in the EO Browser service and manually sandstorm days, which prevent a clear view of the surface.

Immediately adjacent day comparison using the timelapse allows the enhancement of the CH_4_ signal while reducing the surface variability effect. This dynamic method has proven to be the most effective to identify the weakest emissions, which, analyzed individually, would go unnoticed, and, also, to lower the detection limit of S2 to about 1800 kg/h on the most optimal surfaces.

**Figure 2:**
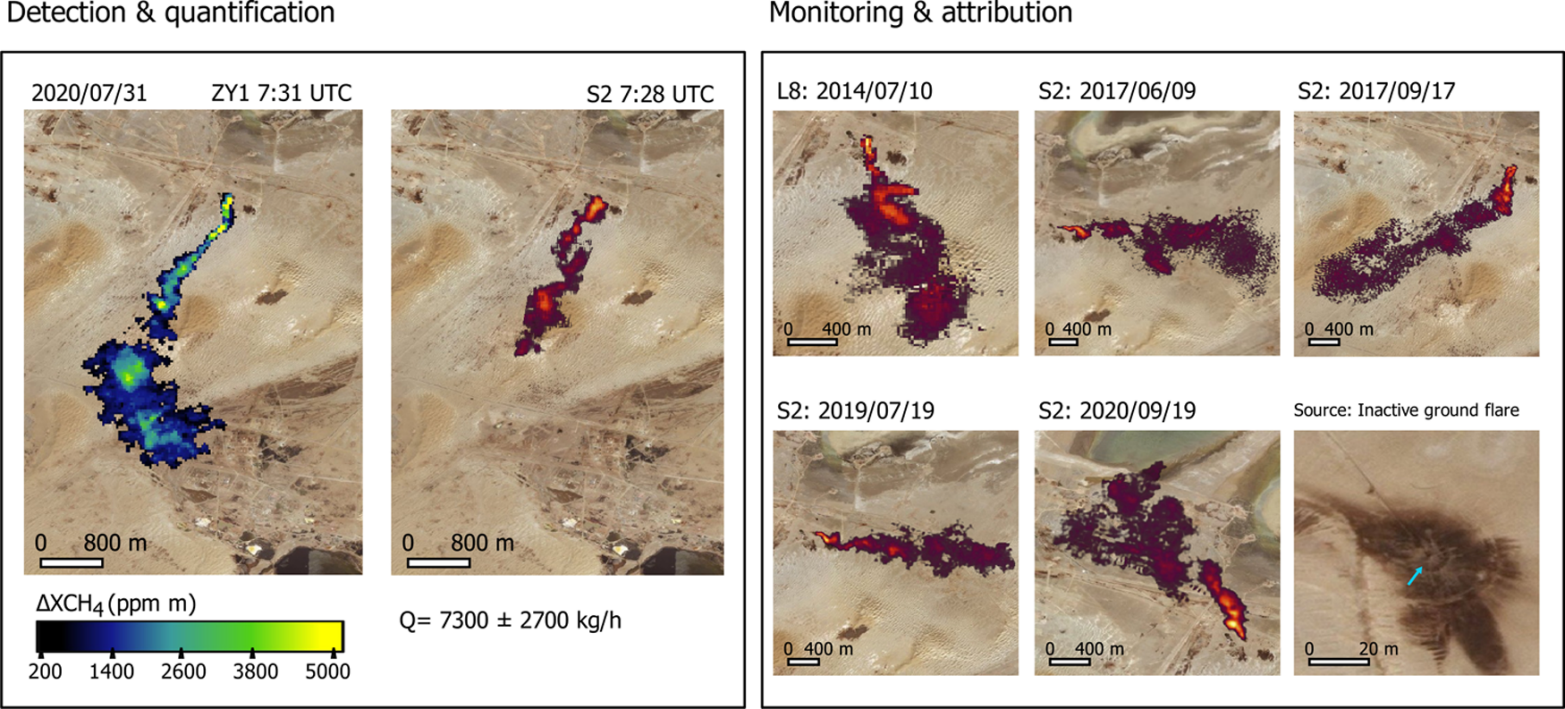
Examples of emissions detected from the A.3 emission point (see Figure 3). Left, plume detected by both ZY1 and S2 within a 3-min time difference. Right, time series of plumes detected at A.3 with the S2 and L8 multispectral satellites. A true-color composite of the emission point, based on visual imagery, is shown in the lower right corner. The background image for all panels is from Bing Maps.

We have obtained the S2 detection figures shown in this paper (Figures 2 and S3) applying the B12 and B11 bands ratio of two contiguous days from the same satellite (S2A or S2B) and with the same orbit whenever possible, as is described in the eq 1.
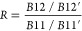
1where *R* is the result of the band ratio, *B*12 and *B*11 are the bands of the emission day, and *B*12′ and *B*11′ are the bands of the nearest clear-sky day observed with the same S2A or S2B satellite from the same viewing on which there is no emission. This method provides the CH_4_ plume avoiding the maximum interference in the signal from other surface components. Also, avoid the increase of noise in the result due to miss-registration and viewing differences.^37^

On the other hand, we have opted for a conservative approach of not quantifying each single emission detected with multispectral systems because of the lower sensitivity of those instruments to methane. A first estimation of the intensity of the emissions is obtained from the scarcer but more accurate hyperspectral measurements.

To observe the area before 2017, we have used Landsat 5 (L5) and 8 multispectral satellites, both with 30 m pixel resolution. We have obtained the results in the same way as S2 but using the B07/B05 band ratio in L5 and B07/B06 in L8. The L8, the overpass time is about 20 min different from ZY1, PRISMA, and S2, so that coincident detections on the same day have not been considered valid for empirical comparison. We have used the entire L5 time series (1984–2012) to observe locations with high emission potential, and the L8 time series (2013-present) to all emitters identified with S2. Both satellites have a revisit cycle of 16 days^38^ (see Table S1 for more information).

### Annual Variability

The SCB of Turkmenistan is a stable and largely homogeneous area, both in surface and climatic terms if we compare it with other parts of the world. Its low precipitation, low cloud cover and high evaporation make it a particularly optimal area for emission detection, also on a temporal scale, where, for example, the surface area does not vary according to vegetation cycles. However, there are still several variables that affect the continuous monitoring of emissions and their measurement.

First, cloudy days and precipitation are not distributed equally throughout the year. The consequence of this variability can be seen in Figure 4, where the number of detected emissions is higher in the months near July, coinciding with the summer season in Turkmenistan (May to September), which is usually hot and dry.^39^ As a result, in this period, we have more clear-sky observations, and the surface remains dry and less variable, which is favorable for emission detection and leads to more emission detections in general. On the other hand, most of the precipitation falls between January and May,^39^ so the number of valid observations is smaller, and the detection of emissions is more complicated if the humidity breaks the homogeneity of the surface.

In this study, we have taken into account the variability in the number of valid observations throughout the year to estimate the difference in the emission number from one year to another. We have calculated the ratio between the number of clear sky days and the number of days with emissions for each year.

On the other hand, other physical variables change throughout the year, such as surface and air temperature, solar angle, or surface albedo, for example, that could affect the detection limit of the sensors. In this study, we have not delved into the impact of these variables. We assume that the detection limit of S2 is close to 1800 kg/h in the best case, based on the empirical estimation of the samples obtained during the study.

### Emitter Identification

The identification of the sources was carried out by inspection of high-resolution visual images from Google Earth, Bing Maps, and ESRI, depending on the acquisition date available for each area on each platform. This has been possible because the 20 and 30 m resolution data provide the source coordinates with sufficient accuracy.

In three cases, we were not able to identify the origin of the emissions due to lack of up-to-date very high-resolution surface imagery (in some southern areas most recent image is from 2015 and Planet’s 3 m/pix images are not enough for these cases) and insufficient geographic information about Turkmenistan’s O&G infrastructure.

Regarding the emitters identified as flares, there is a wide variety of flare systems within the O&G. In Turkmenistan, we have identified emissions from different types of flares, but we refer to all of them as ″flares″ within the study. Additional details on the type of emitting flares are provided in the Supporting Information (Table S2, Materials and Methods Section).

We consulted with technical experts^40^ with field experience on the region to support our identification of the emitters (mainly flares).

### Flaring Signal

Flaring can be detected by satellites with bands in the SWIR, due to the flame’s strong signal in that spectral region, with the emission peak at 1.6 μm.^41^

We have used various satellite data sources to monitor the emitter’s flaring, depending on the period. For 2017–2020, we used S2 data, where the flaring shows an intense signal in the B12 band. For the dates before 2017, we used data from the Landsat series (up to 1984),^42^ the VIIRS historical series (up to 2012) and MODIS (up to 2000) using the Google Earth Engine,^43^ EO Browser,^36^ SkyTruth,^44,45^ and FIRMS^46^ platforms. In some cases, the past flaring activity can be seen in the Google Earth, Bing Maps, and ESRI high-resolution historical imagery (see Figure S5).

## Results

### Analysis of Emission Sources

**Figure 3:**
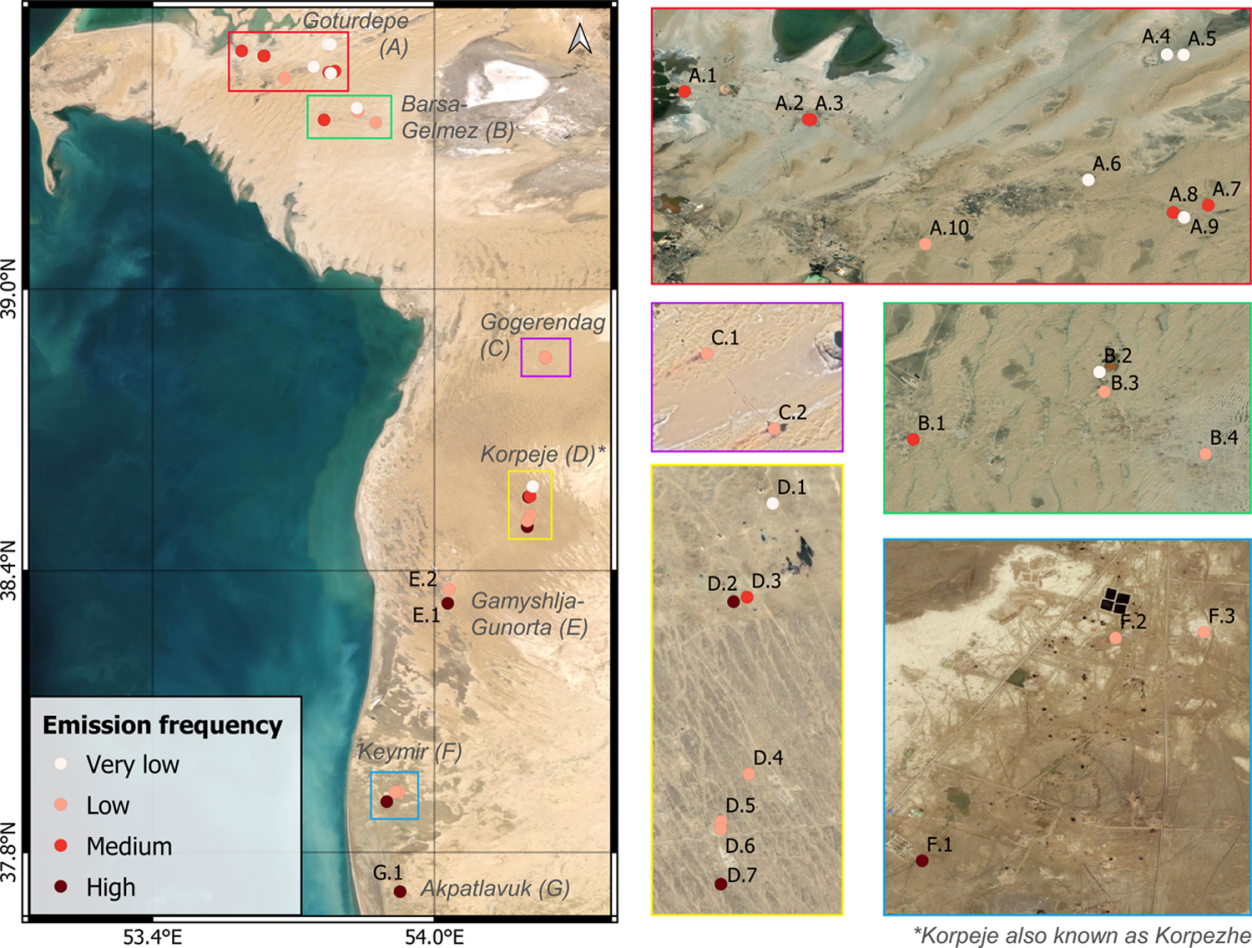
Spatial distribution of point emissions in Turkmenistan’s South Caspian Basin. The emission frequency corresponds to the number of emissions detected by S2 with respect to the number of clear-sky days with S2 overpasses between 2017 and 2020, where “high” represents an emission frequency range 48–37%, “medium” 37–15%, “low” 15–3%, and “very low” 3–1%. Emission points are labeled with alphanumeric codes. Codes with the same letter belong to the same field. The names of the fields are included in the left-hand panel in italics, with the code letters of the emitters in brackets. Background images are extracted from the most recent high-resolution imagery in the ESRI, Google Satellite, or Bing Maps web portals.

Combining the hyperspectral and multispectral high-spatial-resolution satellite data, we have detected 29 emission points with activity between January 2017 and November 2020 (Figure 3). The areas with the highest density of point sources in our high-resolution survey coincide with the strongest CH_4_ enhancements over the west coast of Turkmenistan, as seen in the regional-scale maps generated from TROPOMI moderate resolution data (Figure 1).

The 20–30 m sampling of the hyperspectral and multispectral satellites in combination with very high-resolution imagery from Google Earth, Bing Maps, and ESRI (<2.5 m/pix) provide sufficient information to determine the coordinates of emission sources with high precision, especially for those emitters with many detected plumes (see Figure 2). Combining these data, we have identified the sources of 26 of the 29 points. We find that most of the emitters (24 of them) are inactive flares that vent gas. Several of them had flaring activity before 2017, and three of them had an active flare at the beginning of the study period (Figure S5), followed, afterwards, by episodes of CH_4_ emissions when the flare was no longer active.

The flaring activity is discussed in more detail in the following sections.

The 24 emitting flares are distributed across different onshore fields of the SCB with a higher density in the Goturdepe, Barsa-Gelmez, and Korpeje fields (Figure 3). These three fields have the highest production (Table 1) and are also three of the oldest ones in the basin. This coincides with the 2013 Carbon Limits report, which indicates that most of the flares are concentrated in fields built before 1990.^47^ Most of the emitters are in fields where the predominant activity is crude oil and condensate production, except for the Korpeje field that extracts mainly gas (see Table 1). Two of the emitting flares are around an oil power plant linked to the Goturdepepe field.

**Table 1:** Classification of Oil and Gas Production Fields Where Emissions Have Been Found^a^

field	oil and gas category	production (kbbl/d)			number of emitters	detected emissions				total emissions
		2018	2019	2020		2017	2018	2019	2020	
Goturdepe	crude oil	43.014	30.000	30.137	10	138	50	64	141	393
	condensate	0.001	0.001	0.001						
	NGL	0.060	0.042	0.042						
Barsa-Gelmez	crude oil	28.000	20.000	13.667	4	32	39	23	32	126
	condensate	0.001	0.001	0.059						
	NGL	0.021	0.015	0.029						
Gogerendag	crude oil	0.000	0.000	0.007	2	0	0	3	21	24
	condensate	0.003	0.004	0.009						
Korpeje	crude oil	0.003	0.003	0.046	7	45	25	43	74	187
	condensate	0.002	0.002	0.002						
	NGL	0.160	0.160	0.158						
	gas	18.919	18.919	18.879						
Gamyshlja Gunorta	crude oil	0.004	0.003	0.768	2	7	14	24	28	73
	condensate	0.003	0.003	0.683						
Keymir	crude oil	0.003	0.004	4.648	3	7	17	25	41	90
	condensate	0.001	0.001	4.212						
	NGL	0.028	0.028	0.650						
Akpatlavuk	crude oil	0.004	0.003	0.000	1	21	16	12	2	51
	condensate	0.003	0.003	0.000						
total		90.23	69.19	74.00	28	250	161	194	339	944

a ″Field″ refers to the name of the field; ″Oil and Gas Category″ is the type of production activity in each field; ″Production″ is the amount of production in kbbl/day in the years 2018–2020; ″Number of emitters″ is the number of emitting points that have been found in each field; ″Detected emissions″ is the number of days with emissions that have been observed by year; and ″Total emissions″ is the total number of plumes observed in each field in the entire study period. Oil and Gas category and production data is based on Rystad database.^31^

The fields where we have detected emissions are directly managed by two large state companies, which at the same time control most of the Turkmenistan fields.^31^ Although all SCB fields have been analyzed, no emissions have been recorded between January 2017 and November 2020 from the fields managed by the other five companies operating in the area, which are based in other countries.

The emissions of two other sources, A.10 and E.2 (see Figure 3), are due to pipeline leaks that persist over several months. In the case of A.10, the leak is active for more than a year between 2019 and 2020, while at E.2, we observe emissions from April to October 2018. It has been possible to confirm that these two emissions are due to leaks because the start of the emission coincides with anomalies in the surface (visible in RGB images), and the CH_4_ plumes seem to originate in pipelines. In E.2, it is also possible to see a liquid spill emanating from the leak (see Figure S6).

In the case of the three remaining emission points (A.8, A.9, and B.1), it is difficult to attribute them to a particular source. Leaks are the most likely origin, given that the three points are located just above pipes, that the facilities are old in these fields and that, according to the 2013 Carbon Limits report, the pipeline network (controlled by the national gas company Turkmengas) ″is characterised by its old and inefficient equipment″.^47^ However, we do not have access to records of incidents or leaks recorded by the operators and cannot confirm the source of the emissions because the very high-resolution imagery available is not sufficiently up to date to support this hypothesis, and the resolution of S2 and Landsat imagery is not sufficient in these cases to distinguish a clear change in the surface in visual imagery. Regarding the temporal evolution of these emissions, point A.9 only shows emissions during September 2020, which would indicate either that the emission source has already been fixed or that the emission rates have decreased below the S2 detection limit. Point A.8 shows emissions since 2017, whereas point B.1 has been emitting at least since 2015, according to L8 detections. Both have maintained emissions at least until the end of our study period in November 2020.

None of the detected emitters is linked to mud volcanoes despite those being potential sources of CH_4_ and having a high presence in the area.

### Magnitude of the Emissions

We have developed methods to quantify CH_4_ concentration enhancements and flux rates from the hyperspectral data.^13^ Using the hyperspectral data, we have detected 25 plumes from 12 of the emitters on different dates (see Figure S4). The estimated emission fluxes vary considerably, with 1400 ± 400 kg/h being the lowest emission and 19.600 ± 8000 kg/h the largest detected emission.

The coincident overpass time of S2, PRISMA, and ZY1 (2–5 min difference) has enabled us to capture emissions concurrently with S2 and the hyperspectral systems (see Figures 2 and S3). Using the accurate CH_4_ concentration enhancement maps from the hyperspectral systems as a reference, we can assess the detection limits of the substantially lower signal-to-noise ratio S2 observations. This exercise shows that S2 can detect emissions of at least 1800 ± 200 kg/h for the Turkmenistan desert scenes, as this is the smallest emission for which we have a coincident detection with the hyperspectral data. This is the minimum flux rate that we set for the plumes detected by S2 (944 plumes in total) between January 2017 and November 2020. This detection limit value is slightly lower than that Varon et al.^27^ indicated (∼3000 kg/h) for the most optimal surfaces, as is the case in most of Turkmenistan.

### Temporal Evolution of the Emissions

**Figure 4:**
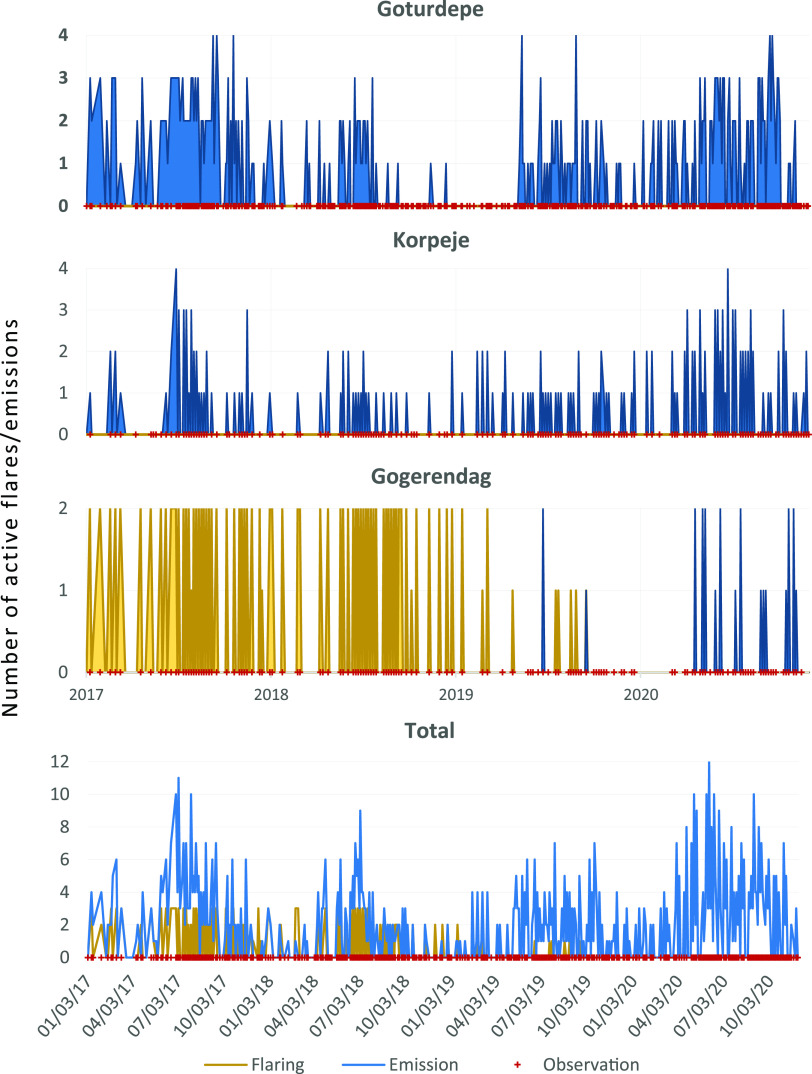
Temporal evolution of emissions in the Goturdepe (A.X), Korpeje (D.X), and Gogerendag (C.X) fields, as well as the daily total number of active emissions detected from the 29 sites found in this study. The vertical axis indicates the number of points that were emitting or flaring at the same time on the same day.

The monitoring of emissions during 2017–2020 using S2 data has shown a remarkable difference in the number of detected plumes from each emitter over time. In general, 2018 was the year with the fewest detected emissions, while 2020 has been the year with the most detected emission plumes, double the number detected in 2018 (see Figure 4 and Table 1). This relationship also holds when we normalize the number of emissions by the number of clear-sky observations in each period.

Not all fields have had the same evolution. Figure 4 shows the examples of the Goturdepe, Korpeje, and Gogerendag fields (Figure 3) as representative cases of different temporal evolution patterns. Goturdepe is one of the fields with the highest number of identified emitters, and its temporal evolution clearly shows a decrease in the number of emissions between 2018 and the beginning of 2019, while in the years 2017 and 2020, the emission density is notably higher. Regarding the Korpeje field, Varon et al. reported in 2019 emissions from three different points,^25^ one of which is named in this paper as D.7. Immediately after the article submission (May 2019) emissions stopped from that source, but both our analysis and the one by Varon et al.^27^ show that emissions resumed after a few months (according to our observations in September 2019, see Figure S7 emitter D.7). Finally, the Gogerendag field stands out for the direct relationship between the end of the use of flaring and the start of emissions, i.e., at the beginning of the monitoring period, emitters in this field had flaring activity, but CH_4_ emission events began to occur after the flaring signal was no longer visible. This same flaring-emission relationship is repeated at point F.3, which shows an intense flaring signal at the beginning of the study, but in July 2018, the flaring disappears. In July 2019, CH_4_ emissions start to be observed intermittently until the end of the study period.

Analyzing the emitters individually, we also see that there is wide variability in their emitting frequency. Of the 29 points, 6 show emissions on only between 1 and 3% of the observed clear-sky days, i.e., they rarely present emissions above our 1800 kg/h detection limit. On the opposite side, 5 points show emissions in more than 38% of the observed days. The low frequency emissions could be explained by emergencies or well purging, that are very unusual events, and where the law allows the venting of large amounts of gas from flaring systems for a short period. However, the more frequent emitters would conflict with the ″Rules for the Development of Hydrocarbon Fields″ of the Turkmen law, which bans continuous gas flaring and venting.^47^ Detailed information on the frequency of emissions is provided in Table S2, and the temporal evolution of each emitter is provided in Figure S7.

We also look at the emissions of the region before our 2017–2020 core study period. First, the longer time series of L8 satellite data reveal that at least 15 of the 29 emitters identified in the study period were already emitting large amounts of CH_4_ before January 2017, as shown in Figure 2 (first window, right-hand-side panel) and Figure S8. Second, the SCIAMACHY sensor onboard ENVISAT^48^ also provides information on the history of emissions in the area, in this case, at the regional scale. Comparing the distribution of our single detections with the regional XCH_4_ map from TROPOMI (Figures 1 and 3), we can infer that the CH_4_ enhancement observed by TROPOMI in the northern part of the study area is the result of many moderate to high-frequency emitters, while in the south the areas of CH_4_ enhancement are related to one or a few very high-frequency emitters (Figure S9). This relationship holds in older data from SCIAMACHY. Between 2003 and 2010 SCIAMACHY already observed a higher CH_4_ concentration in the northern area of the SCB, over the Goturdepe and Barsa-Gelmez fields and another hot spot over the Korpeje and Gamyshlja Gunorta fields but did not observe a CH_4_ enhancement over the southernmost Keymir and Akpatlavuk fields. The year of the facility installation coincide whit the data, where most of the emitters in the first four fields already existed before 2010, but the higher frequency emitters in the southern fields (F.1 and G.1) are later, according to Landsat images. So, these points did not contribute to the average result of the data collected by SCIAMACHY (Figure S9), but they are reflected in the TROPOMI dataset.

Furthermore, L5 historical data reveal that this type of emissions has been present in these fields since, at least, 1987. We show some examples in Figure S10.

### Flaring

According to VIIRS data, flaring has been progressively decreasing in fields where we have identified the emitters since 2016. For example, the flare volume in 2020 was more than 90% lower than in 2012 (Figure S11). At the state level, the trend has been the same until 2019, where the flare volume has continuously decreased since VIIRS records have been kept, and in 2019 it is almost half of what it was in 2012 (2.42 billion cubic meters in 2012 and 1.34 billion cubic meters in 2019). In 2020 the decreasing trend changed, and the volume of gas flared at the state level increased, but according to our analysis, this occurred only in fields where mainly gas is extracted, and especially in the those located in the west of Turkmenistan, in the Amu Darya basin.^44^

As we previously discussed, several of the CH_4_ emitters detected in our survey follow this trend of flaring reduction. C.1, C.2, and F.3 have flaring activity at the beginning of the monitoring but then change from flaring to gas emission. In addition, we have observed that at least six other emitters had an active flame in the past, but vented gas later (Figure S5). The fact that several of the emitters currently venting CH_4_ showed flaring activity in the past suggests a relationship between the decrease in flaring at the expense of an increase of venting.

## Discussion

In this study, we have used a combination of satellites to produce a large-scale survey of individual CH_4_ emitters active between 2017 and 2020 on the west coast of Turkmenistan, one of the world’s largest CH_4_ hotspot regions. First, areas of interest within the region have been identified using medium-resolution data from TROPOMI. Two types of high-resolution data have then been used to detect, quantify, and monitor the activity of the identified 29 strong CH_4_ emitters over time. Hyperspectral satellites have mapped plumes with fluxes between 1400 ± 400 and 19,600 ± 8100 kg/h, which indicates that the emissions from Turkmenistan are often extremely high; the S2 multispectral satellite has enabled the systematic monitoring of emissions above 1800 kg/h, showing an increase in the number of detections in 2020 compared to the previous years, and the longer time series of the L5 and L8 missions (1984–2012 and 2013–today, respectively) have shown that several emitters have been venting CH_4_ for decades.

Our analysis reveals that the large amounts of CH_4_ emitted in this region are mainly due to the venting of gas from oil fields. We find that venting is related to the decrease in the use of flaring as a method to treat excess gas. This points to the risks of penalizing flaring without effective measures to control venting. Secondly, the emissions not related to venting are linked to the pipelines, which have gas leaks during long time periods. Identifying these high emitting sources is fundamental for any short-term mitigation strategy, as efficiently detecting and fixing them can significantly reduce emissions.

High-resolution satellites capable of detecting CH_4_ emissions, in combination with mid-resolution satellites with daily global coverage such as TROPOMI and its successor Sentinel-5 instruments, bring a new era in the monitoring of industrial emissions, both locally and globally, with the potential to provide early warnings in near real-time. In addition to the already operational high-resolution satellites (GHGSat, PRISMA, ZY1, S2, and Landsat), new missions such as MethaneSAT, EMIT, Carbon Mapper, EnMAP, CHIME, and SBG are expected to reinforce possible monitoring systems even further.

## Supporting Information Available

Additional background data, satellite data and sensor information, complementary information about detected emissions and emission sources, including imagery, and detailed information about emitters (PDF)
