# Supplementary material for: Satellites Detect Abatable Super-Emissions in One of the World’s Largest Methane Hotspot Regions
Source: Environ Sci Technol. 2022 Feb 1;56(4):2143–52. doi: 10.1021/acs.est.1c04873 (PMC9940854; doi:10.1021/acs.est.1c04873)
Supplement: Supplementary file 1 — es1c04873_si_001.pdf [file es1c04873_si_001.pdf]

Satellites detect abatable super-emissions in one of the world's largest methane hotspot regions

Itziar Irakulis-Loitxate[1], Luis Guanter[1], Joannes D. Maasakkers[2], Daniel Zavala-Araiza[3,4], Ilse Aben[2]*

[1]Research Institute of Water and Environmental Engineering (IIAMA), Universitat Politècnica de València (UPV), 46022, Valencia, Spain.

[2]SRON Netherlands Institute for Space Research, 3584 CA, Utrecht, The Netherlands.

[3]Environmental Defense Fund, Reguliersgracht 79, 1017 LN Amsterdam, The Netherlands.

[4]Institute for Marine and Atmospheric Research Utrecht, Utrecht University, 3584 CC Utrecht, The Netherlands.

* Corresponding author email: iiraloi@doctor.upv.es

Summary:

Number of pages: 20

Number of figures: 11

Number of tables: 2

Section 1. Supporting figures and additional information

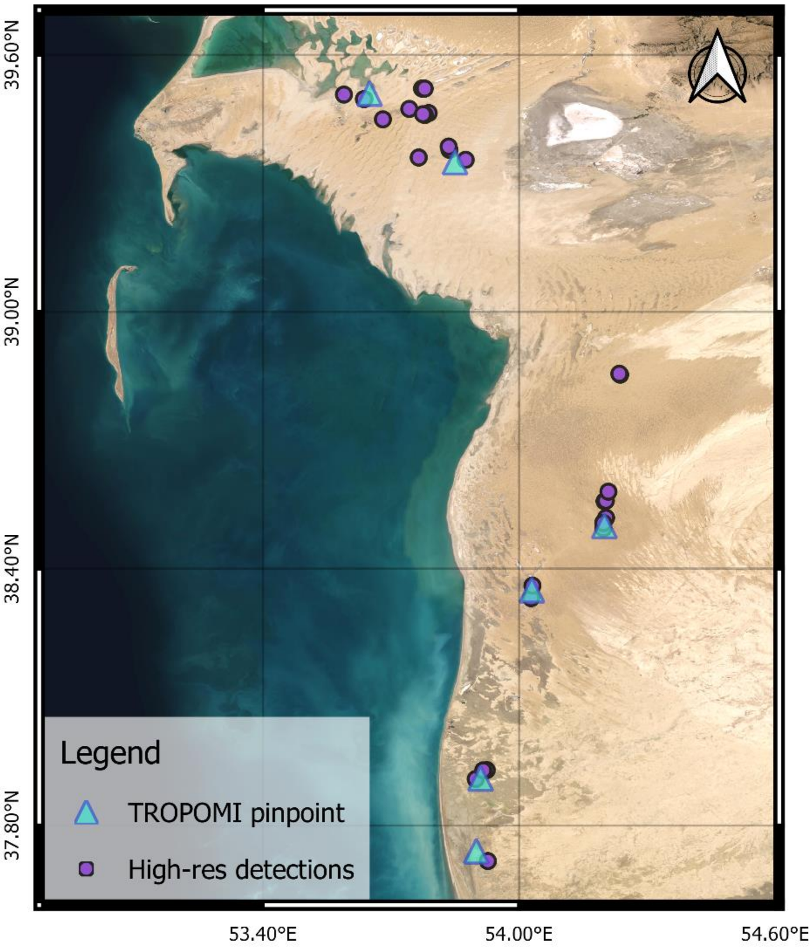

Figure S1. The locations pinpointed by TROPOMI (blue triangles), and the emitter points (purple circles) found in the study.

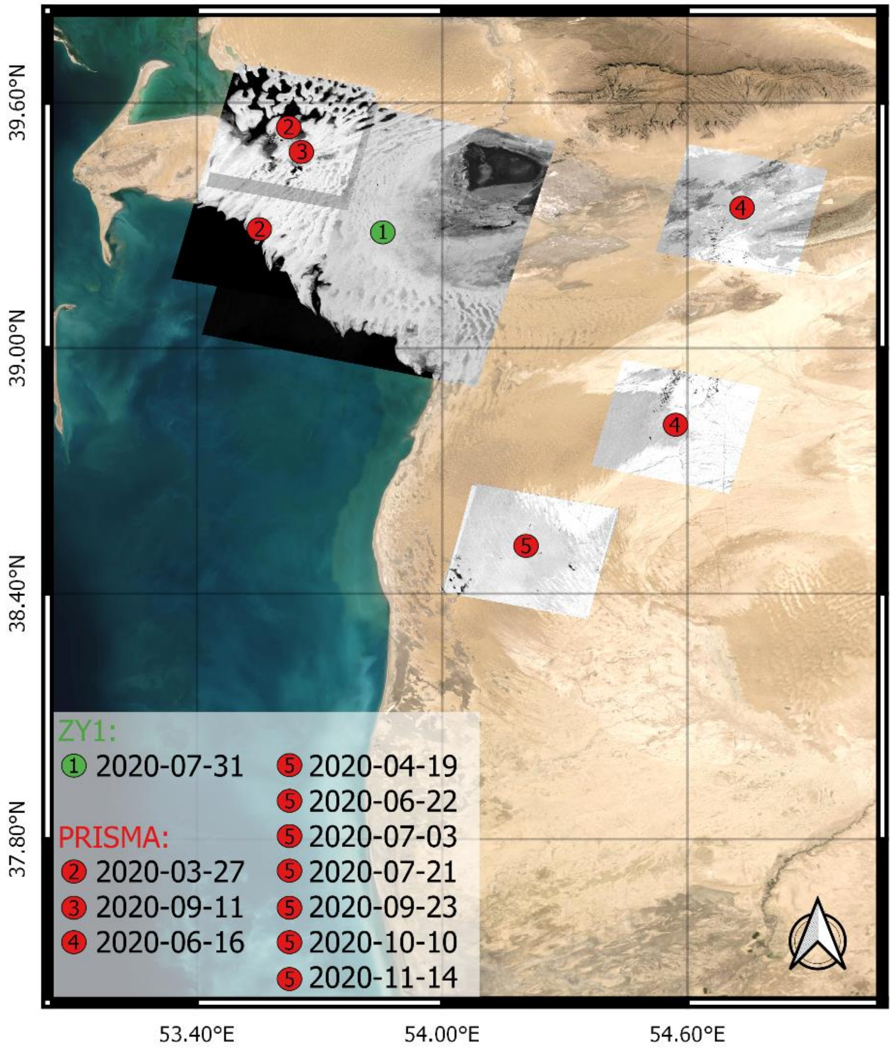

Figure S2. Spatial coverage of ZY1 and PRISMA hyperspectral data used in this work. The Chinese ZY1 mission was launched in September 2019 and has onboard the AHSI sensor whose images cover a 60X60 km^2 area, while the Italian PRISMA mission, launched in March 2019, provides images with 30X30 km^2 coverage. Both missions have a spatial resolution of 30 m. The hyperspectral acquisition requests were first made with a focus on the key points identified by TROPOMI, and then those were extended to the rest of key areas as O&G extraction fields, pipeline crossings, flares that in the past had shown an active flame, and mud volcanoes. Due to the difficulty to obtain data from these sensors

in the short term, we could not cover some areas in that time range. Many PRISMA images have been acquired from the catalogue, while others have been obtained based on requests for targeted locations.

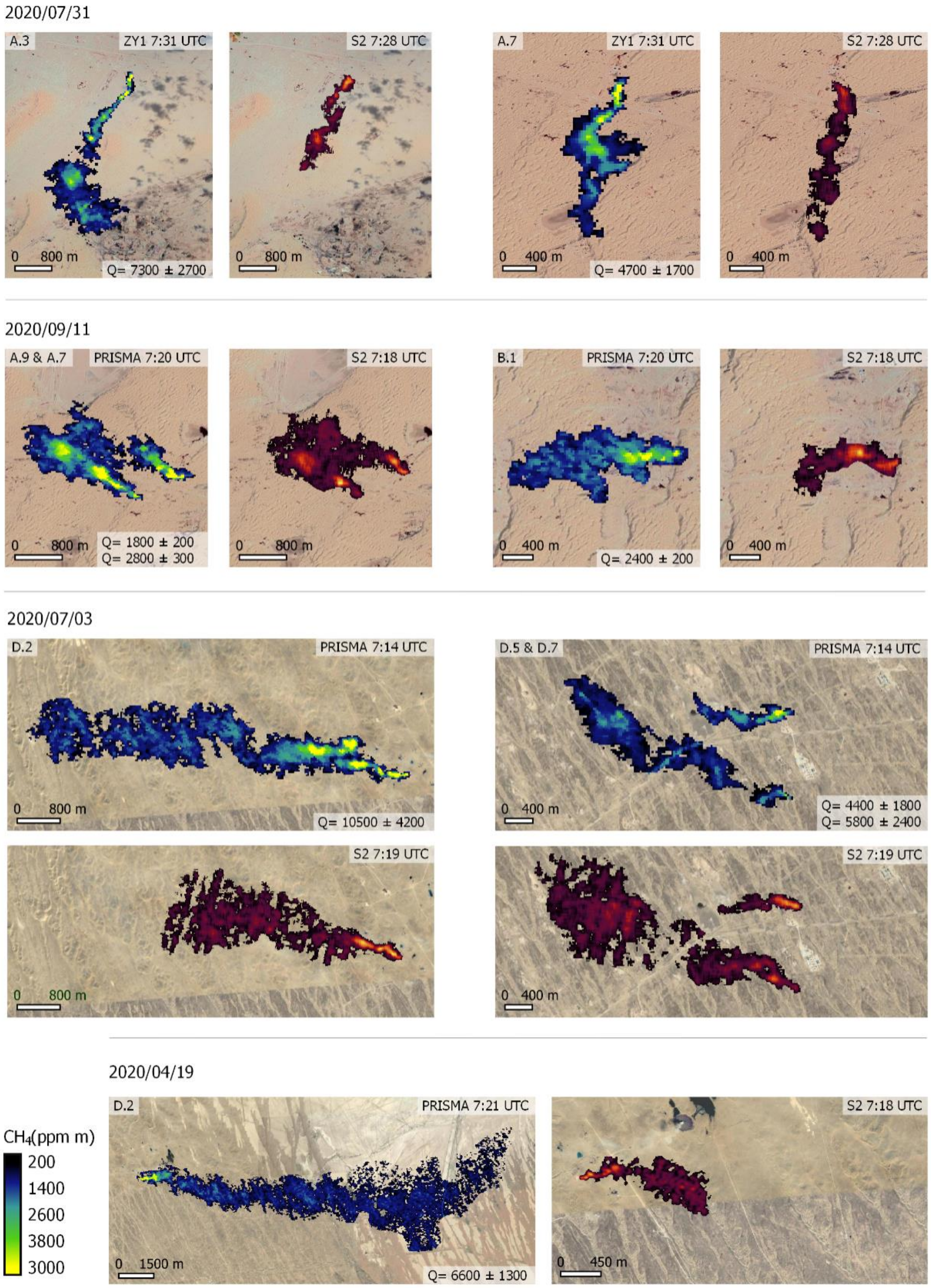

Figure S3. Simultaneous detections of Sentinel 2 (S2) CH_4 plumes with PRISMA and ZY1 satellites within minutes of each other.

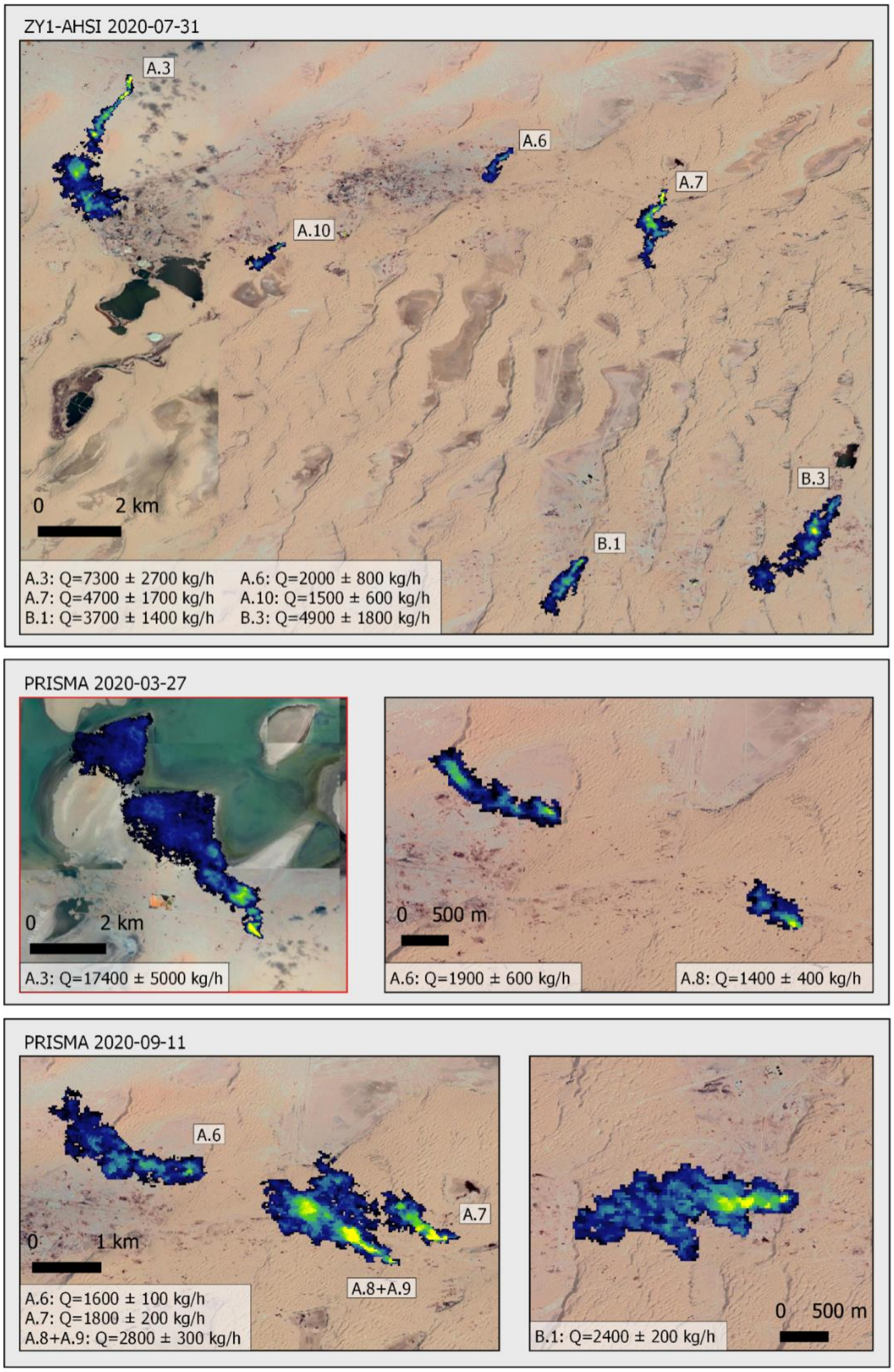
ZY1-AHSI 2020-07-31
A.3
A.6
A.7
A.10
B.3
B.1
0 2 km
A.3: Q=7300 ± 2700 kg/h
A.7: Q=4700 ± 1700 kg/h
B.1: Q=3700 ± 1400 kg/h
A.6: Q=2000 ± 800 kg/h
A.10: Q=1500 ± 600 kg/h
B.3: Q=4900 ± 1800 kg/h
PRISMA 2020-03-27
0 2 km
A.3: Q=17400 ± 5000 kg/h
0 500 m
A.6: Q=1900 ± 600 kg/h
A.8: Q=1400 ± 400 kg/h
PRISMA 2020-09-11
A.6
A.7
A.8+A.9
0 1 km
A.6: Q=1600 ± 100 kg/h
A.7: Q=1800 ± 200 kg/h
A.8+A.9: Q=2800 ± 300 kg/h
0 500 m
B.1: Q=2400 ± 200 kg/h

38

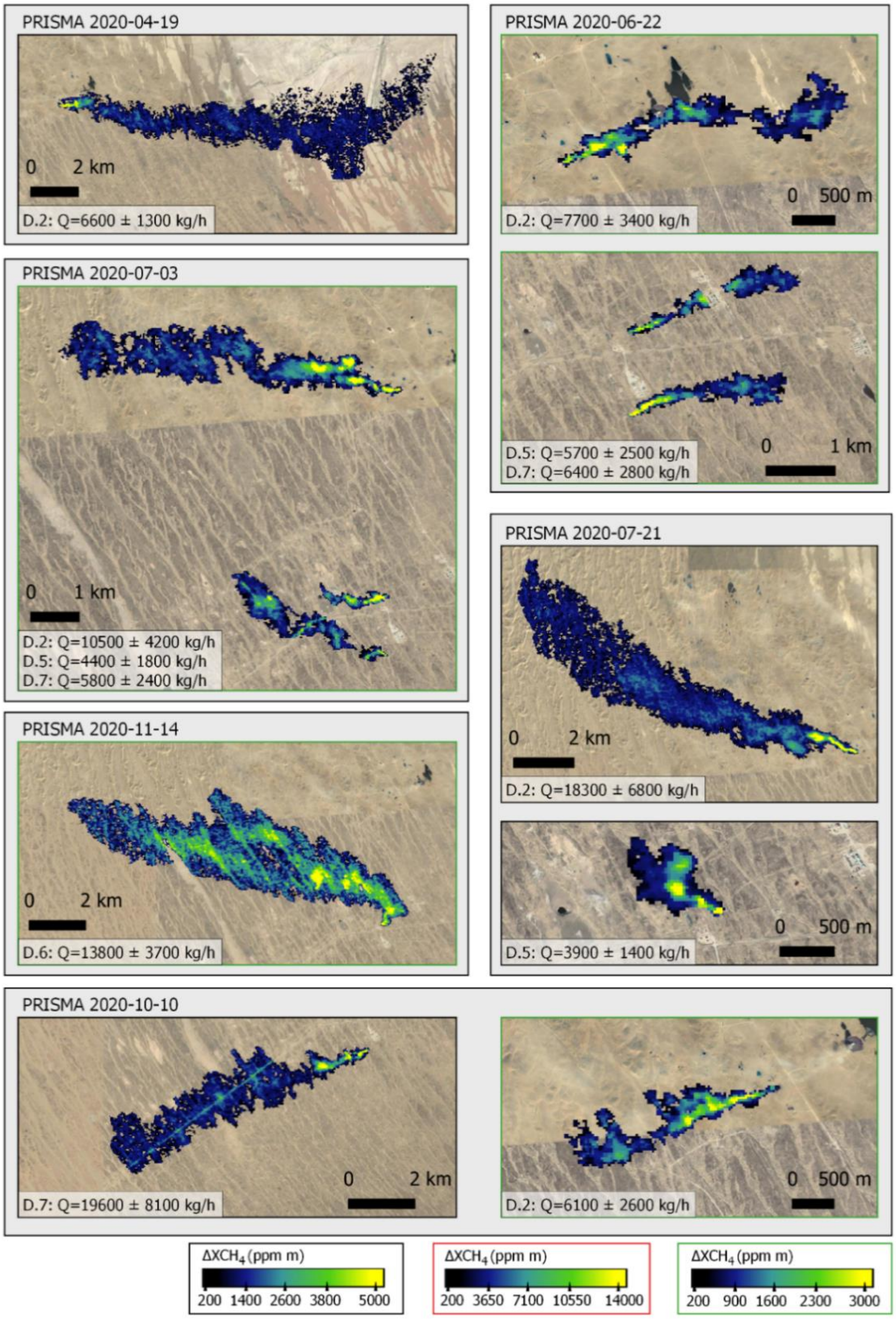

Figure S4. All CH_4 plumes detected with the ZY1 and PRISMA hyperspectral satellites in the survey period. The color scale corresponding to each plume is indicated with the color of the map outline (black, red, or green).

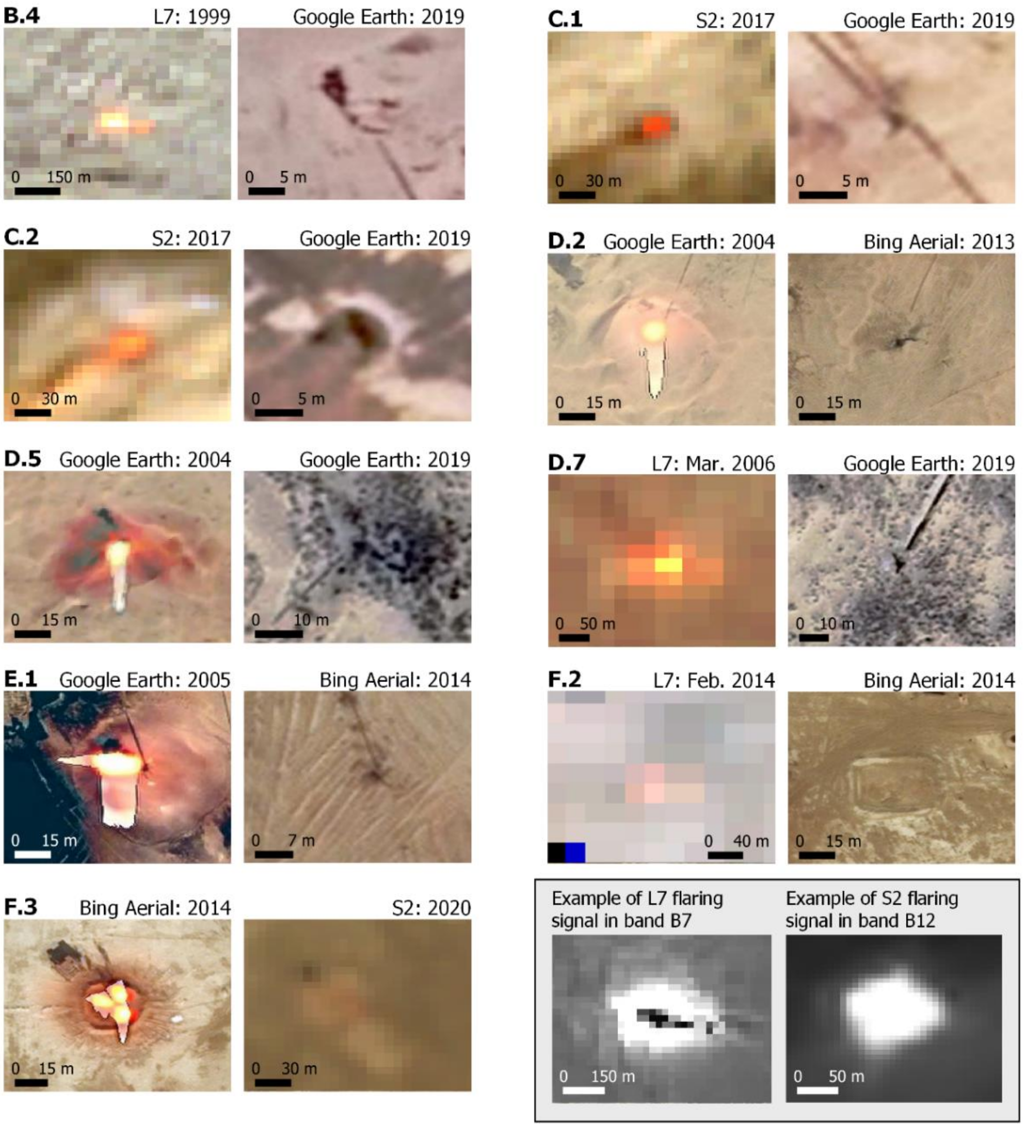

Figure S5. Flares with active flaring in the past and current inactive appearance seen in RGB. Bottom right two examples of active flares as seen in the Landsat 7 (L7) B7 and S2 B12 bands (points D.7 and C.2 respectively), i.e. in the CH_4 absorption bands. In the Landsat B7 and S2 B12 bands, the CH_4 absorbs the signal (low values), while the flaring emits a very high signal (very high values) compared to the surface.

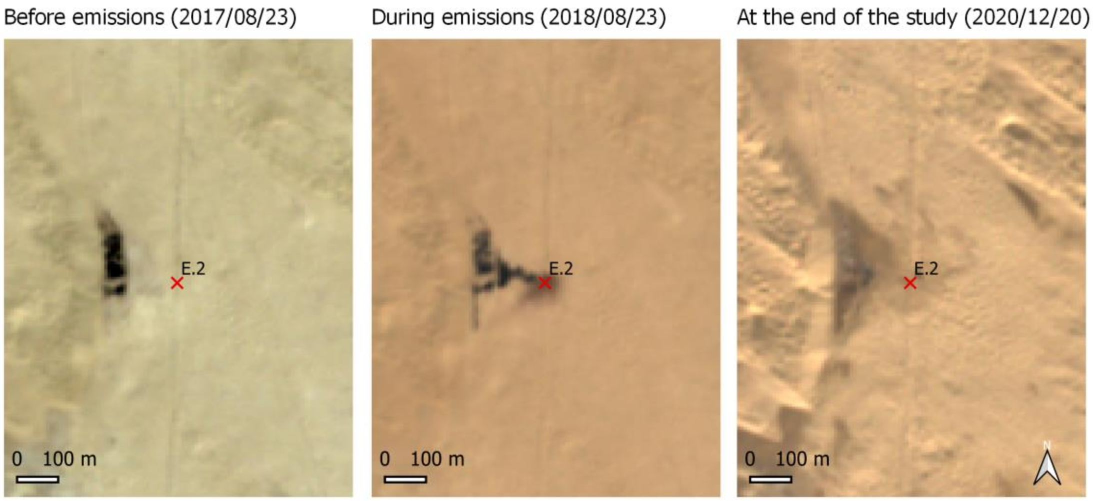

Figure S6. The evolution of the E.2 emission point seen in RGB before, during and after the emissions derived from a leak. During the emission period a black liquid emanating from the emission point is visible.

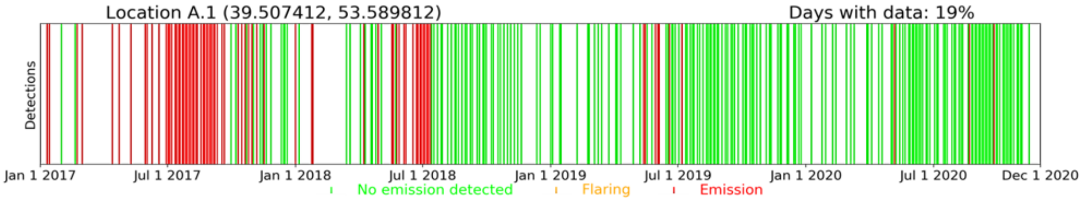
Location A.1 (39.507412, 53.589812)
Days with data: 19%
Detections
Jan 1 2017
Jul 1 2017
Jan 1 2018
Jul 1 2018
Jan 1 2019
Jul 1 2019
Jan 1 2020
Jul 1 2020
Dec 1 2020
No emission detected
Flaring
Emission

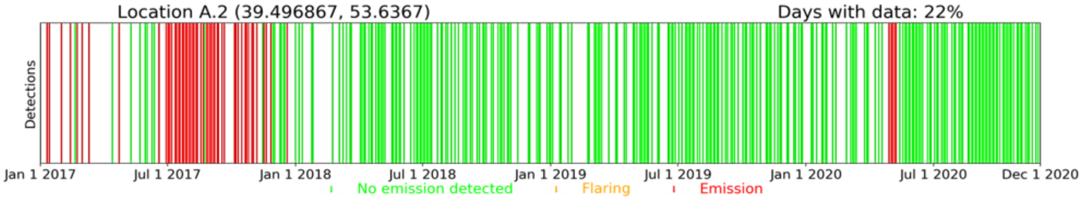
Location A.2 (39.496867, 53.6367)
Days with data: 22%
Detections
Jan 1 2017
Jul 1 2017
Jan 1 2018
Jul 1 2018
Jan 1 2019
Jul 1 2019
Jan 1 2020
Jul 1 2020
Dec 1 2020
No emission detected
Flaring
Emission

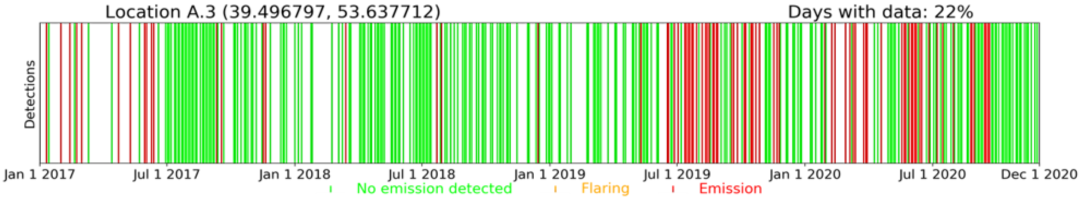
Location A.3 (39.496797, 53.637712)
Days with data: 22%
Detections
Jan 1 2017
Jul 1 2017
Jan 1 2018
Jul 1 2018
Jan 1 2019
Jul 1 2019
Jan 1 2020
Jul 1 2020
Dec 1 2020
No emission detected
Flaring
Emission

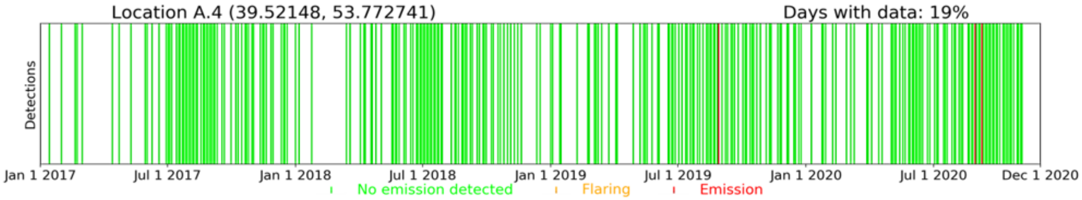
Location A.4 (39.52148, 53.772741)
Days with data: 19%
Detections
Jan 1 2017
Jul 1 2017
Jan 1 2018
Jul 1 2018
Jan 1 2019
Jul 1 2019
Jan 1 2020
Jul 1 2020
Dec 1 2020
No emission detected
Flaring
Emission

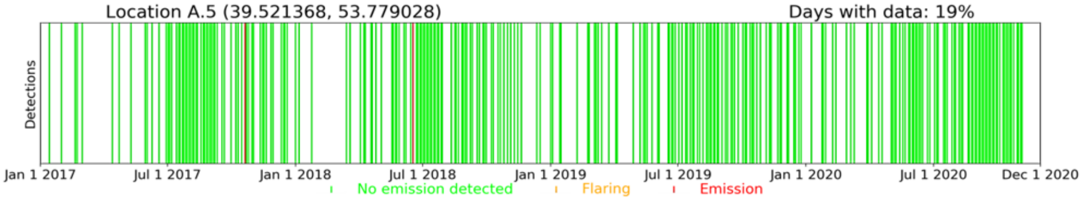
Location A.5 (39.521368, 53.779028)
Days with data: 19%
Detections
Jan 1 2017
Jul 1 2017
Jan 1 2018
Jul 1 2018
Jan 1 2019
Jul 1 2019
Jan 1 2020
Jul 1 2020
Dec 1 2020
No emission detected
Flaring
Emission

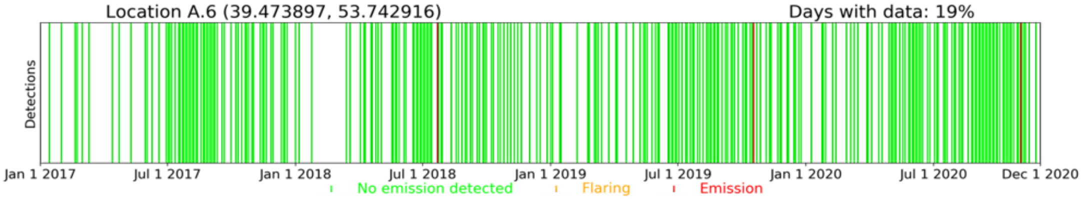
Location A.6 (39.473897, 53.742916)
Days with data: 19%
Detections
Jan 1 2017
Jul 1 2017
Jan 1 2018
Jul 1 2018
Jan 1 2019
Jul 1 2019
Jan 1 2020
Jul 1 2020
Dec 1 2020
No emission detected
Flaring
Emission

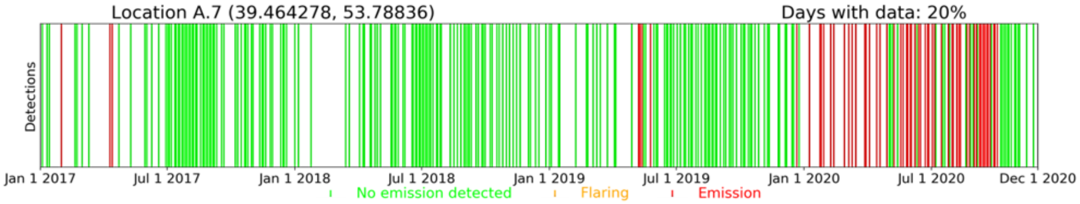
Location A.7 (39.464278, 53.78836)
Days with data: 20%
Detections
Jan 1 2017
Jul 1 2017
Jan 1 2018
Jul 1 2018
Jan 1 2019
Jul 1 2019
Jan 1 2020
Jul 1 2020
Dec 1 2020
No emission detected
Flaring
Emission

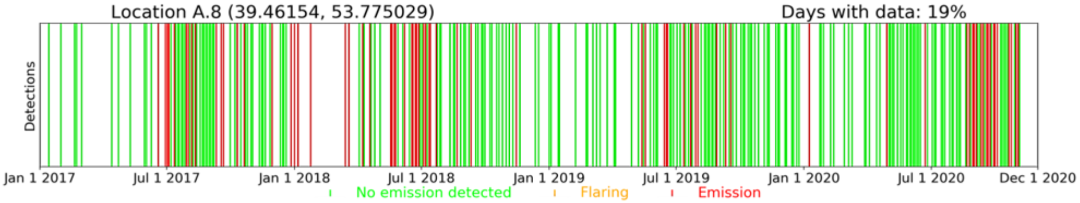
Location A.8 (39.46154, 53.775029)
Days with data: 19%
Detections
Jan 1 2017
Jul 1 2017
Jan 1 2018
Jul 1 2018
Jan 1 2019
Jul 1 2019
Jan 1 2020
Jul 1 2020
Dec 1 2020
No emission detected
Flaring
Emission

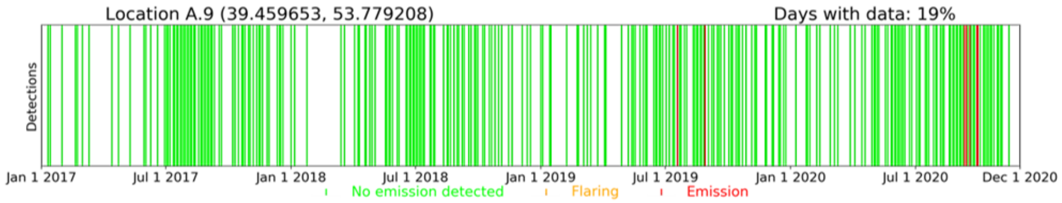
Location A.9 (39.459653, 53.779208)
Days with data: 19%
Detections
Jan 1 2017 Jul 1 2017 Jan 1 2018 Jul 1 2018 Jan 1 2019 Jul 1 2019 Jan 1 2020 Jul 1 2020 Dec 1 2020
No emission detected Flaring Emission

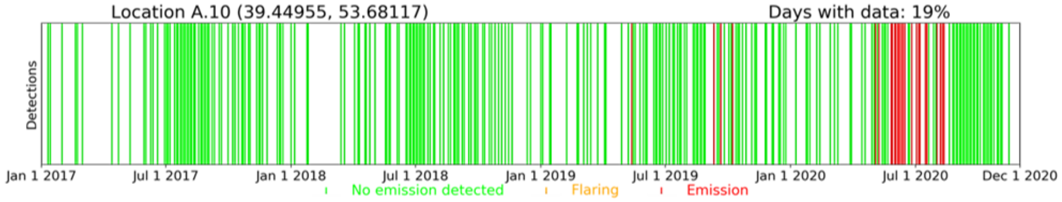
Location A.10 (39.44955, 53.68117)
Days with data: 19%
Detections
Jan 1 2017 Jul 1 2017 Jan 1 2018 Jul 1 2018 Jan 1 2019 Jul 1 2019 Jan 1 2020 Jul 1 2020 Dec 1 2020
No emission detected Flaring Emission

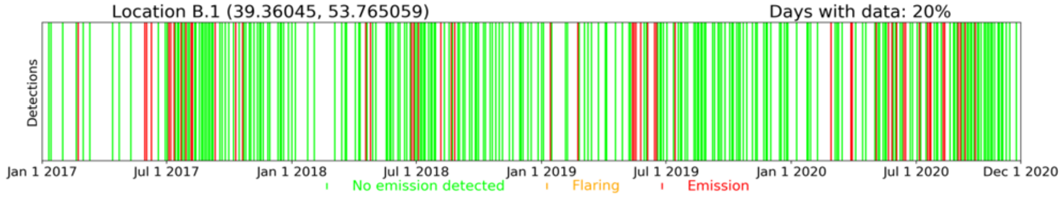
Location B.1 (39.36045, 53.765059)
Days with data: 20%
Detections
Jan 1 2017 Jul 1 2017 Jan 1 2018 Jul 1 2018 Jan 1 2019 Jul 1 2019 Jan 1 2020 Jul 1 2020 Dec 1 2020
No emission detected Flaring Emission

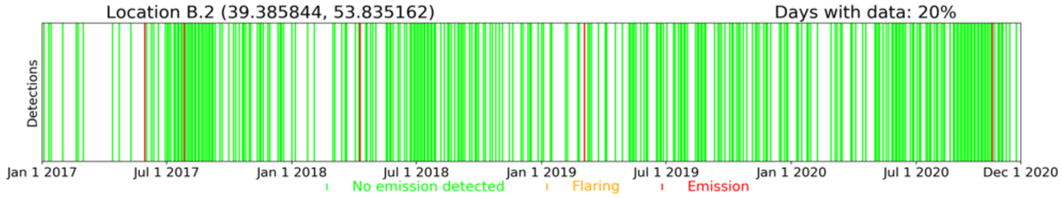
Location B.2 (39.385844, 53.835162)
Days with data: 20%
Detections
Jan 1 2017 Jul 1 2017 Jan 1 2018 Jul 1 2018 Jan 1 2019 Jul 1 2019 Jan 1 2020 Jul 1 2020 Dec 1 2020
No emission detected Flaring Emission

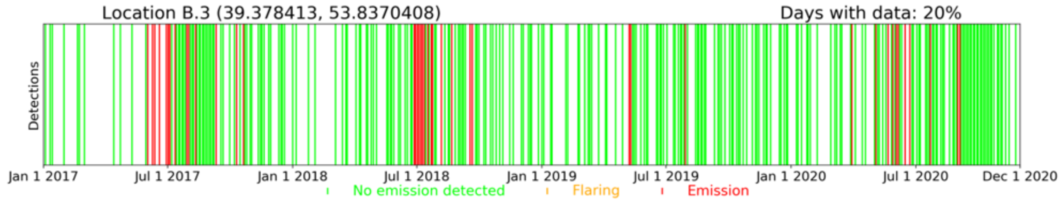
Location B.3 (39.378413, 53.8370408)
Days with data: 20%
Detections
Jan 1 2017 Jul 1 2017 Jan 1 2018 Jul 1 2018 Jan 1 2019 Jul 1 2019 Jan 1 2020 Jul 1 2020 Dec 1 2020
No emission detected Flaring Emission

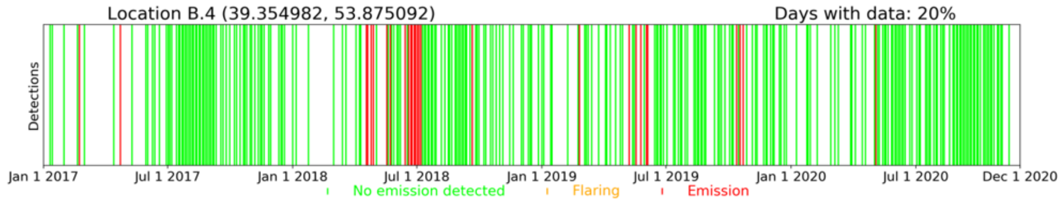
Location B.4 (39.354982, 53.875092)
Days with data: 20%
Detections
Jan 1 2017 Jul 1 2017 Jan 1 2018 Jul 1 2018 Jan 1 2019 Jul 1 2019 Jan 1 2020 Jul 1 2020 Dec 1 2020
No emission detected Flaring Emission

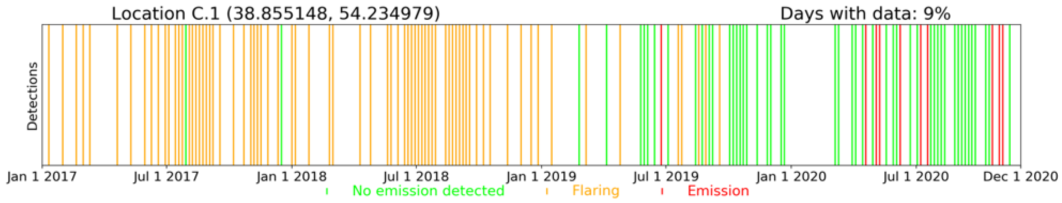
Location C.1 (38.855148, 54.234979)
Days with data: 9%
Detections
Jan 1 2017 Jul 1 2017 Jan 1 2018 Jul 1 2018 Jan 1 2019 Jul 1 2019 Jan 1 2020 Jul 1 2020 Dec 1 2020
No emission detected Flaring Emission

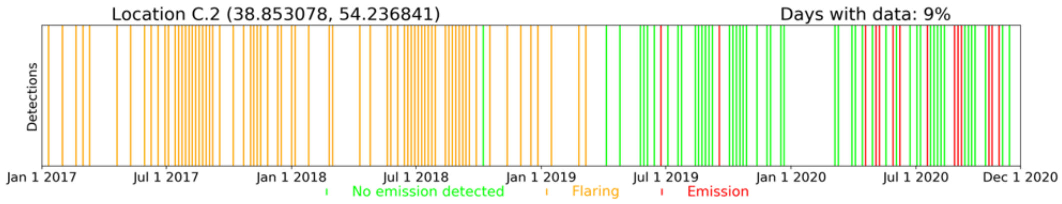
Location C.2 (38.853078, 54.236841)
Days with data: 9%
Detections
Jan 1 2017 Jul 1 2017 Jan 1 2018 Jul 1 2018 Jan 1 2019 Jul 1 2019 Jan 1 2020 Jul 1 2020 Dec 1 2020
No emission detected Flaring Emission

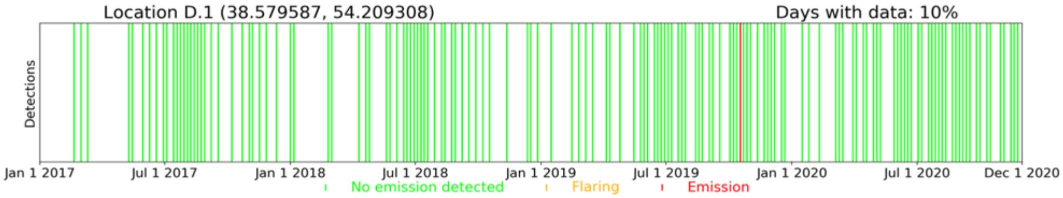
Location D.1 (38.579587, 54.209308)
Days with data: 10%
Detections
Jan 1 2017
Jul 1 2017
Jan 1 2018
Jul 1 2018
Jan 1 2019
Jul 1 2019
Jan 1 2020
Jul 1 2020
Dec 1 2020
No emission detected
Flaring
Emission

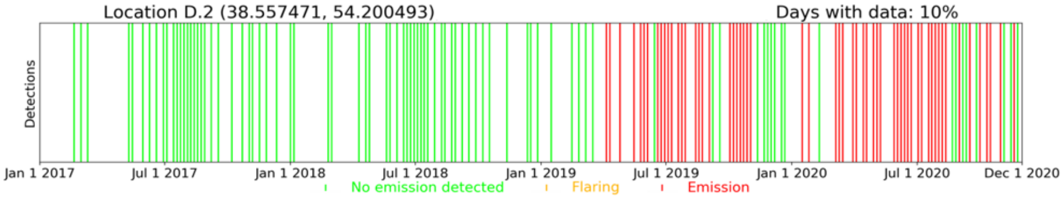
Location D.2 (38.557471, 54.200493)
Days with data: 10%
Detections
Jan 1 2017
Jul 1 2017
Jan 1 2018
Jul 1 2018
Jan 1 2019
Jul 1 2019
Jan 1 2020
Jul 1 2020
Dec 1 2020
No emission detected
Flaring
Emission

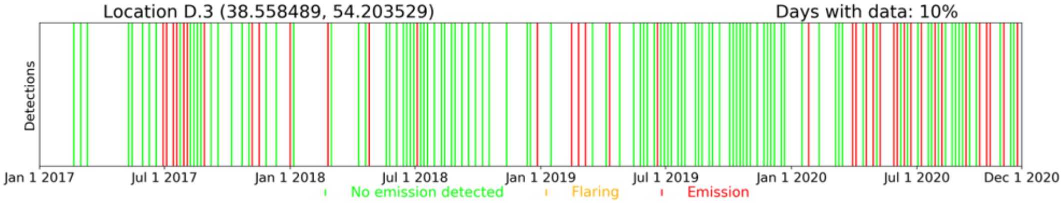
Location D.3 (38.558489, 54.203529)
Days with data: 10%
Detections
Jan 1 2017
Jul 1 2017
Jan 1 2018
Jul 1 2018
Jan 1 2019
Jul 1 2019
Jan 1 2020
Jul 1 2020
Dec 1 2020
No emission detected
Flaring
Emission

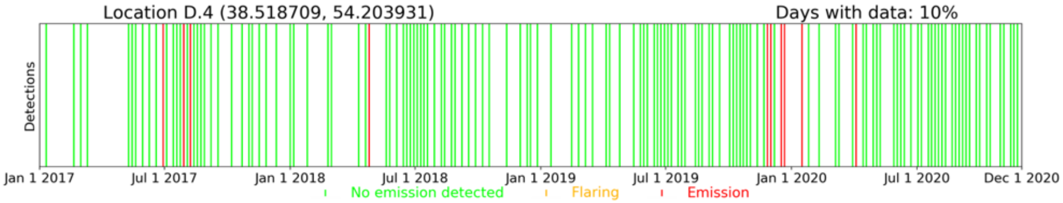
Location D.4 (38.518709, 54.203931)
Days with data: 10%
Detections
Jan 1 2017
Jul 1 2017
Jan 1 2018
Jul 1 2018
Jan 1 2019
Jul 1 2019
Jan 1 2020
Jul 1 2020
Dec 1 2020
No emission detected
Flaring
Emission

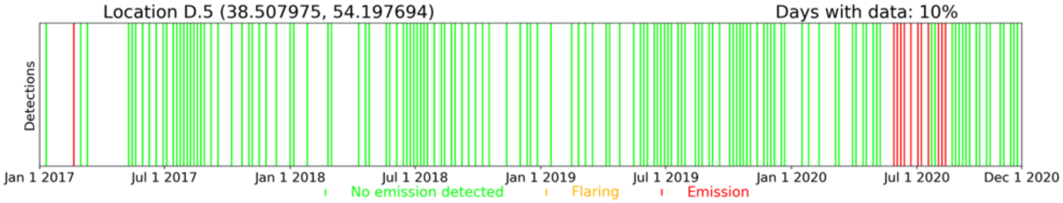
Location D.5 (38.507975, 54.197694)
Days with data: 10%
Detections
Jan 1 2017
Jul 1 2017
Jan 1 2018
Jul 1 2018
Jan 1 2019
Jul 1 2019
Jan 1 2020
Jul 1 2020
Dec 1 2020
No emission detected
Flaring
Emission

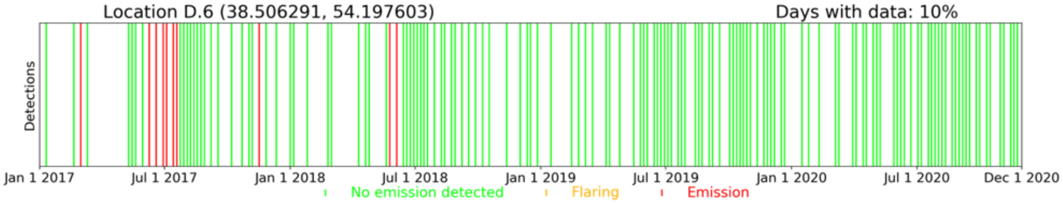
Location D.6 (38.506291, 54.197603)
Days with data: 10%
Detections
Jan 1 2017
Jul 1 2017
Jan 1 2018
Jul 1 2018
Jan 1 2019
Jul 1 2019
Jan 1 2020
Jul 1 2020
Dec 1 2020
No emission detected
Flaring
Emission

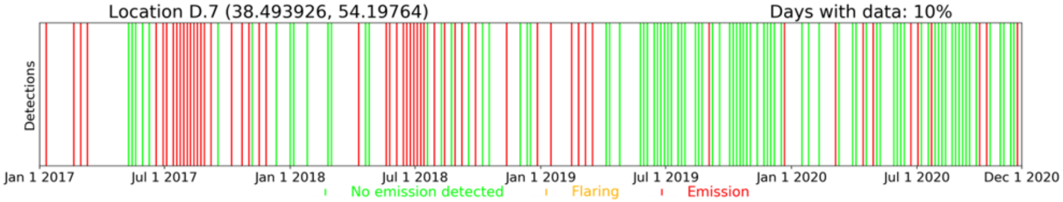
Location D.7 (38.493926, 54.19764)
Days with data: 10%
Detections
Jan 1 2017
Jul 1 2017
Jan 1 2018
Jul 1 2018
Jan 1 2019
Jul 1 2019
Jan 1 2020
Jul 1 2020
Dec 1 2020
No emission detected
Flaring
Emission

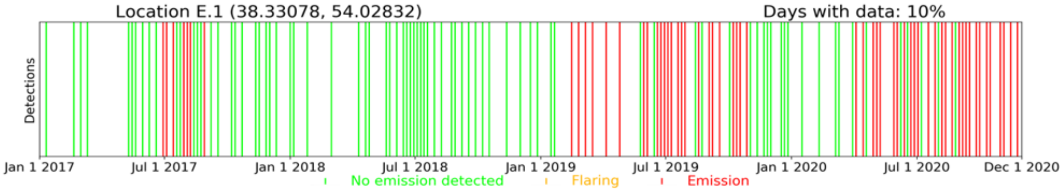
Location E.1 (38.33078, 54.02832)
Days with data: 10%
Detections
Jan 1 2017
Jul 1 2017
Jan 1 2018
Jul 1 2018
Jan 1 2019
Jul 1 2019
Jan 1 2020
Jul 1 2020
Dec 1 2020
No emission detected
Flaring
Emission

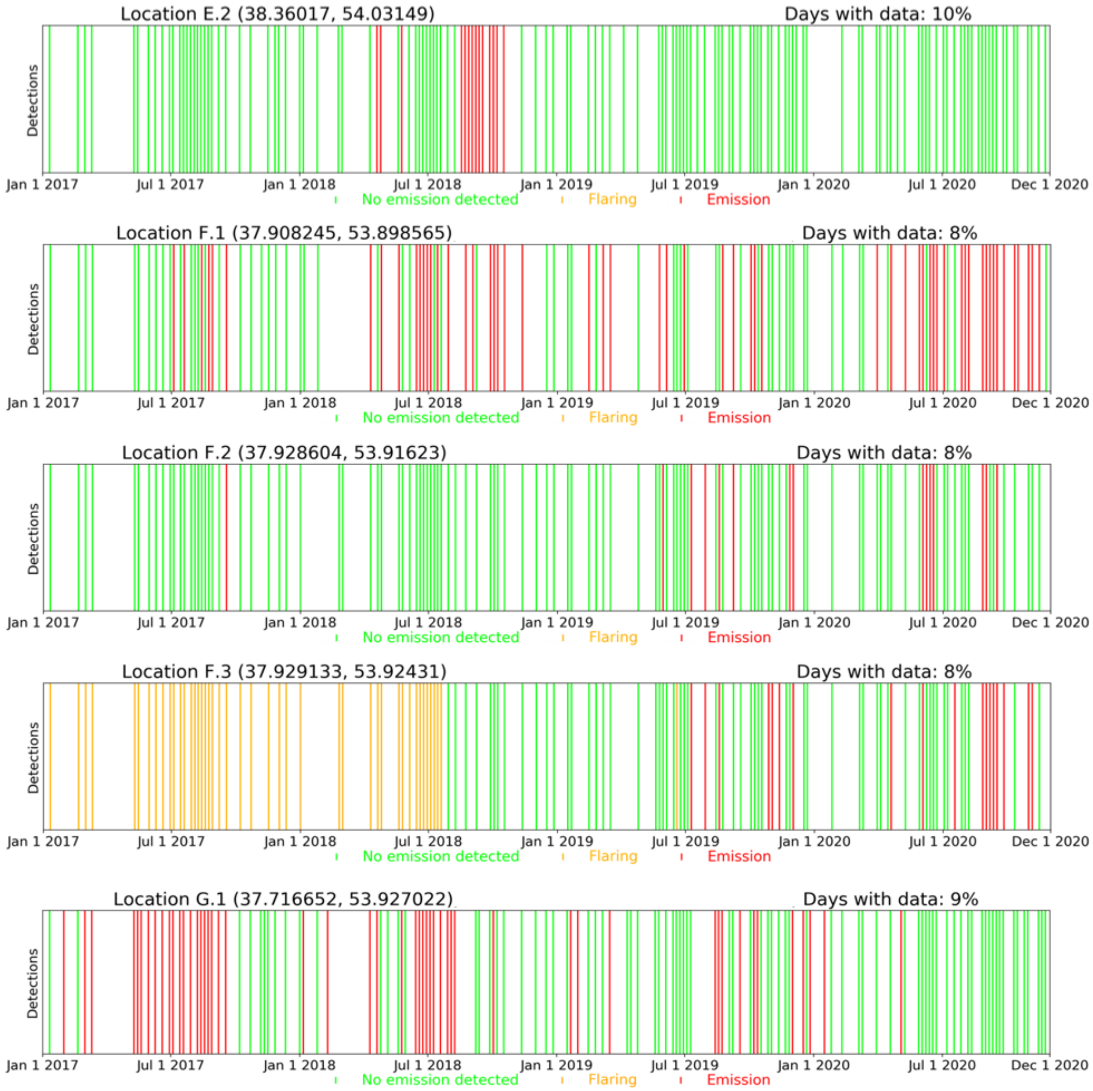

Figure S7. The temporal evolution of the 29 emitters identified during 2017-2020 with S2, where green lines indicate no emission day, red lines indicate emission, and yellow lines indicate active flaring. Cloudy sky days are not included in the series. The Goturdepe (A.X) and Barsa-Gelmez (B.X) emitters contain double data days because two S2 orbits overlap in that area.

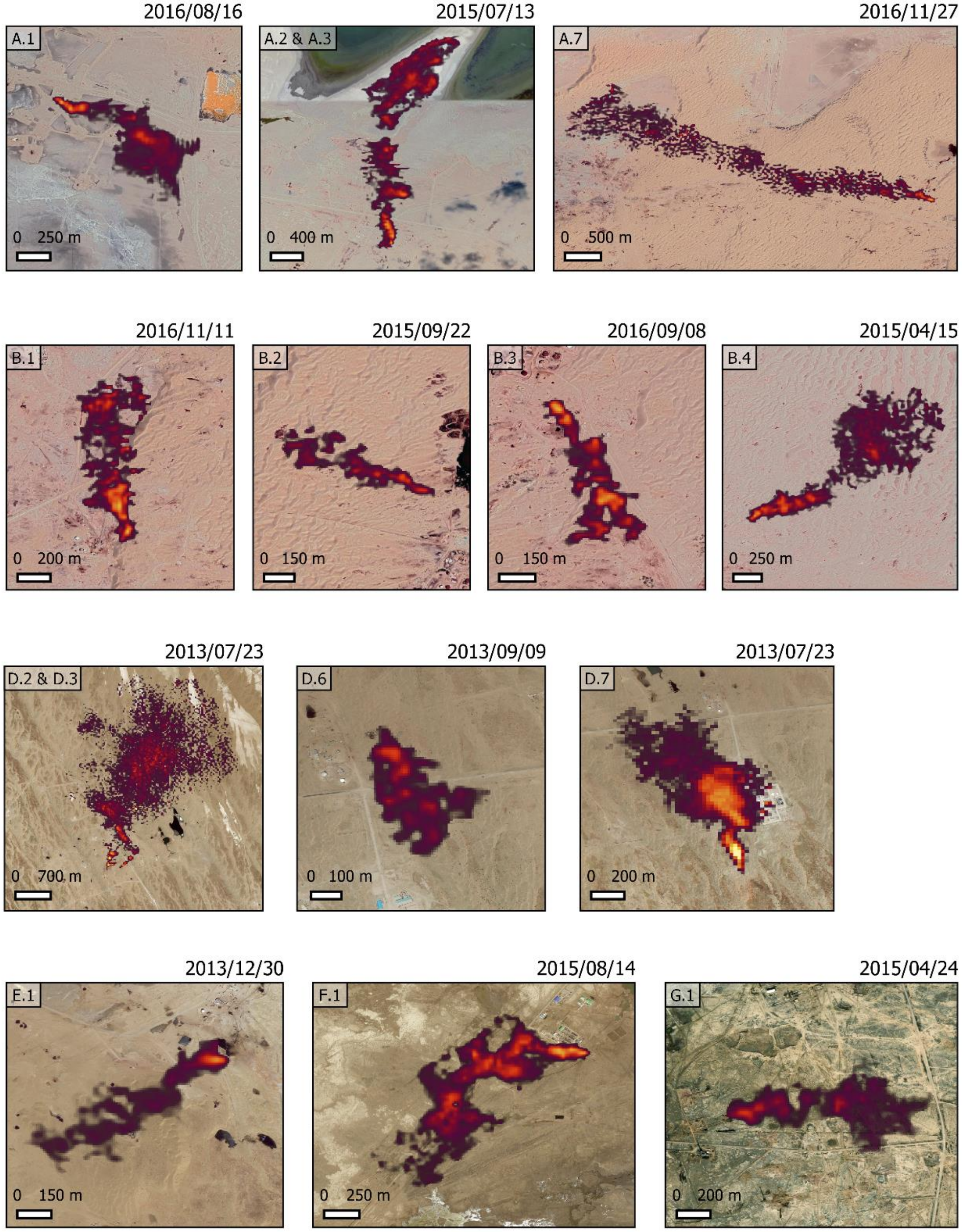

Figure S8. Some Landsat 8 detections from sources that record emissions prior to Sentinel 2 monitoring period.

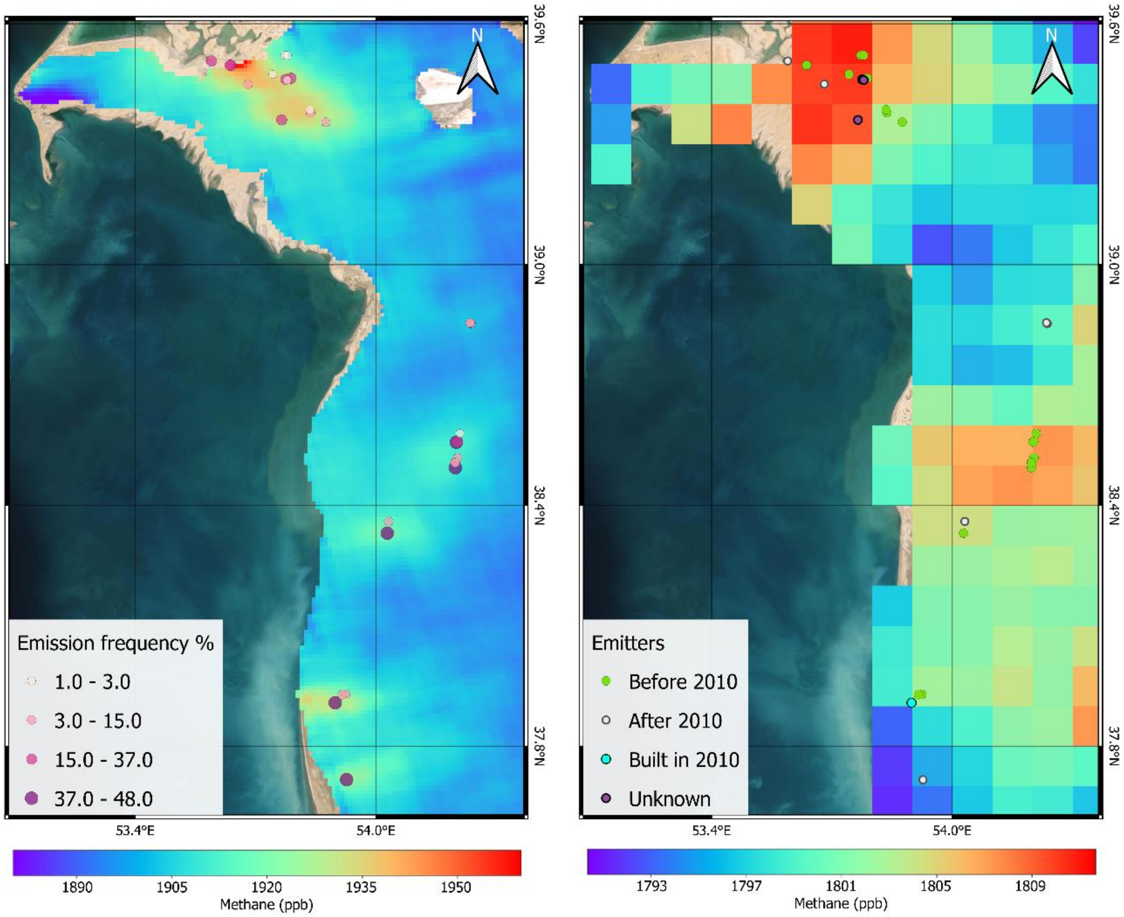

Figure S9. Combination of moderate and low-resolution data from TROPOMI and SCIAMACHY sensors respectively with the emitter points indicated. On the left, the oversampled TROPOMI data between 2018 and 2020 combined with the emitters represented in terms of emission frequency. On the right the SCIAMACHY data oversampled to a 0.1º x 0.1º grid between 2003 and 2010 combined with the emitters found in this study classified according to their possible contribution to the SCIAMACHY data, i.e., whether the emitter existed before 2010 (it could have contributed to the CH_4 enhancement), post-2010 (it could not have contributed), undefined (unidentified emitters) or if it was constructed just in 2010 (it existed in the SCIAMACHY observation period but its contribution should be minimal).

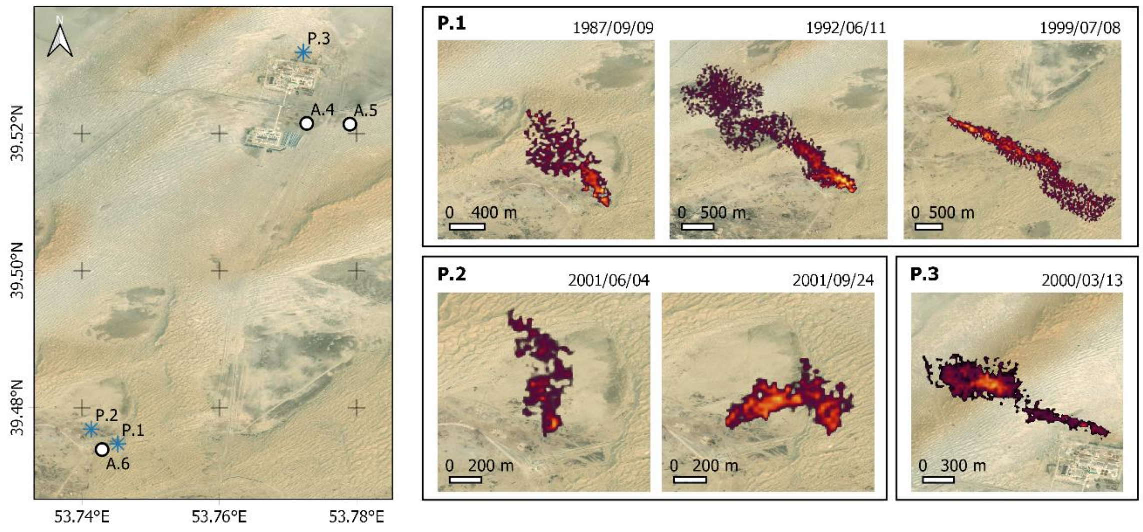

Figure S10. Examples of plume detections in the Goturdepe field using historical data from the L5 multispectral satellite mission. On the left, the general map showing the location of P.1, P.2 and P.3 emitters, which were active during the L5 monitoring period, and the nearby emitters (A.4, A.5, and A.6) active during the S2 monitoring period. On the right, some of the detected plumes from P.1, P.2 and P.3. The first (P.1) is located about 200 m from source A.6 and records emissions from 1987 to, at least, 1999 very frequently. Two years after the last observed emission from P.1, the P.2 emitter, about 350 m north of A.6, began emitting continuously from June through, at least, September 2001. Finally, the third source (P.3) is 1.15 km from emitter A.4, and we have only identified one emission in the Landsat searches. As we do not have very high-resolution data for these dates, nor detailed information about the infrastructure, we have not attributed these emissions to any specific infrastructure. The background image of all panels is from ESRI Satellite.

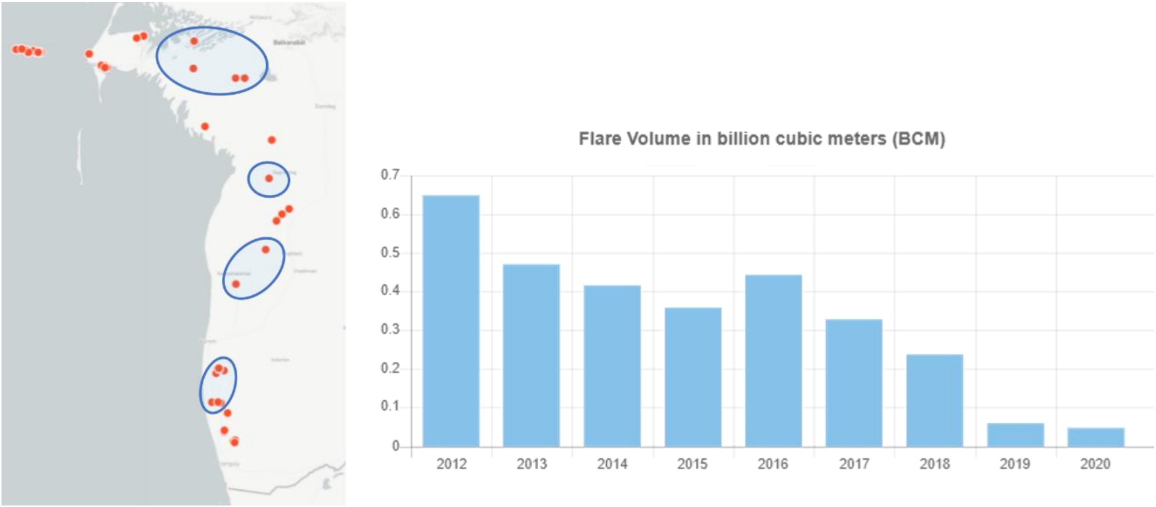

Figure S11. VIIRS detected flaring over the years. On the left, inside the blue polygons, the areas where we have identified emitters, with the points where VIIRS detected flaring between 2012 and 2020. On the right the total flared gas volume in those areas according to VIIRS records each year. These data have been obtained from SkyTruth's Annual Flare Volume map[1].

Section 2. Supporting tables and additional information about flares

Table S1. Satellite characteristics. Where "Spatial resolution" is the pixel size in the images of each satellite; the "Instrument type" column classifies each satellite/sensor between hyperspectral (hundreds of spectral bands), high spectral resolution hyperspectral (hundred high-resolution spectral bands), and multispectral (less than ~20 bands); "Spatial coverage" is the ground area that could cover each orbit or image acquisition; "temporal resolution" is the minimum time period between one acquisition to another of the same place; "CH4 detection capability" classify this set of satellites in terms of their capability to detect CH4 emissions being the better the one with more "+"; and "Period of operation" is the duration of their life.

Satellite/ Sensor	Spatial resolution	Instrument type	Spatial coverage	Temporal resolution	CH4 detection capability	Period of operation
**Sentinel-5P / TROPOMI**	7.0 km x 7.0 km at beginning of mission 7.0 km x 5.5 km since 6 Aug. 2019	High spectral resolution hyperspectral	~2600 km swath	Daily global coverage	+++++	May 2018 - present
**Envisat / SCIAMACHY**	30 km X 60 km	High spectral resolution hyperspectral	960 km swath	Global coverage in 6 days	++++	Mar. 2002 – Apr. 2012
**ZY1 / AHSI**	30 m x 30 m	Hyperspectral	60 km x 60 km	Upon request	++	Sep. 2019 - present
**PRISMA**	30 m x 30 m	Hyperspectral	30 km x 30 km	Upon request	++	Mar. 2019 - present
**Sentinel-2**	20 m x 20 m	Multispectral	290 km swath	10 days each single satellite / 5 days the combined constellation	+	Jun. 2015. Full global coverage since Mar. 2017
**Landsat 8**	30 m x 30 m	Multispectral	185 km x 180 km scene size	16-days	+	Feb. 2013 - present
**Landsat 5 TM**	30 m x 30 m	Multispectral	170 km x 183 km scene size	16-days	+	Mar. 1984 – Jan. 2013

Table S2. Emissions point list. Where "Point ID" is the identifying name assigned to this study. Lat and Long coordinates of the emitter. "Emitter" the type of emitter or source. "O. E. %" is Observed emission %, that is, the percentage of clear-sky days with emissions above the detection limit of S2, and this data is used throughout the document to refer to the emission frequency. "Field" field where it is located.

Point ID	Lat	Long	Emitter	O.E.%	Field
A.1	39.50741	53.58981	Ground flare	29	Goturdepe
A.2	39.49687	53.6367	Ground flare	20	Goturdepe
A.3	39.4968	53.63771	Ground flare	29	Goturdepe
A.4	39.52148	53.77274	Pit flare	1	Goturdepe
A.5	39.52137	53.77903	Ground flare	1	Goturdepe
A.6	39.4739	53.74292	Ground flare	1	Goturdepe
A.7	39.46428	53.78836	Pit flare	21	Goturdepe
A.8	39.4616	53.77502	Undefined	27	Goturdepe
A.9	39.45965	53.77921	Undefined	3	Goturdepe
A.10	39.44955	53.68117	Pipeline	9	Goturdepe
B.1	39.36045	53.76506	Undefined	18	Barsa-Gelmez
B.2	39.38584	53.83516	Ground flare	2	Barsa-Gelmez
B.3	39.37841	53.83704	Ground flare	14	Barsa-Gelmez
B.4	39.35498	53.87509	Ground flare	10	Barsa-Gelmez
C.1	38.85515	54.23498	Ground flare	7	Gogerendag
C.2	38.85308	54.23684	Ground flare	10	Gogerendag
D.1	38.57959	54.20931	Ground flare	1	Korpeje
D.2	38.55747	54.20049	Ground flare	41	Korpeje
D.3	38.55849	54.20353	Pit flare	26	Korpeje
D.4	38.51871	54.20393	Ground flare	7	Korpeje
D.5	38.50798	54.19769	Ground flare	8	Korpeje
D.6	38.50629	54.1976	Ground flare	7	Korpeje
D.7	38.49393	54.19764	Ground flare	39	Korpeje
E.1	38.33078	54.02832	Ground flare	42	Gamyshlja Gunorta
E.2	38.36017	54.03149	Pipeline	10	Gamyshlja Gunorta
F.1	37.90825	53.89857	Elevated flare	48	Keymir
F.2	37.9286	53.91623	Pit flare	12	Keymir
F.3	37.92913	53.92431	Pit flare	15	Keymir
G.1	37.71665	53.92702	Pit flare	38	Akpatlavuk

Flares classification

In the main text, all gas combustion devices are referred to as flares. Flaring systems allow a safe elimination of gaseous waste, provided that the combustion is properly done[2]. However, there is a wide variety of flare types in the O&G sector. Their characteristics depend on multiple factors such as calorific power of the burning fuel, physical state (gas, liquid, or mixture), pressure, flow, geographic location for the population or other activities, availability of land for the installations, or economic availability, among others[2,3].

High-resolution satellite imagery does not provide sufficient information to make a detailed distinction between flare types, but it does allow differentiation between the two main types of flares: elevated flares and ground flares, which are the most visually distinct. Among the ground flares, we can distinguish a third type of flare, known as pit flares.

The principal difference between the two main types of flares (elevated and ground) is the height of the installation. Elevated flares are typically several hundred meters high to reduce plant noise and safety risks but can be more expensive to maintain. Ground flares are ground-level devices that do not require a chimney or supports which, can reduce maintenance costs, although they are more costly to purchase and install. Ground flares can handle larger quantities of gas but present safety risks[3].

Pit flares are ground-level flares in a shallow pool area, surrounded by a dike wall. Pit flares are designed to burn liquid or gaseous waste mainly in emergencies rather than for continuous combustion, and they are used in unpopulated areas to meet safety standards[2].

As indicated in Table S1, we have detected emissions from all three types of flares in Turkmenistan, mainly from ground flares.

REFERENCES

(1) SkyTruth | Flaring Volume Estimates https://viirs.skytruth.org/apps/heatmap/flarevolume.html (accessed Mar 5, 2021).

(2) Bahadori, A. Blow-Down and Flare Systems. *Nat. Gas Process.* **2014**, 275–312. https://doi.org/10.1016/B978-0-08-099971-5.00006-4.

(3) Viaches, D.; Charlton, C.; Kaplan, K. Visual Encyclopedia of Chemical Engineering - Flares https://encyclopedia.che.engin.umich.edu/Pages/EnergyTransfer/Flares/Flares.html (accessed Jul 15, 2021).
